# A new class of protein biomarkers based on subcellular distribution: application to a mouse liver cancer model

**DOI:** 10.1038/s41598-019-43091-z

**Published:** 2019-05-06

**Authors:** Tatjana Sajic, Rodolfo Ciuffa, Vera Lemos, Pan Xu, Valentina Leone, Chen Li, Evan G. Williams, Georgios Makris, Amir Banaei-Esfahani, Mathias Heikenwalder, Kristina Schoonjans, Ruedi Aebersold

**Affiliations:** 1 Department of Biology, Institute of Molecular Systems Biology, ETH Zurich, Zurich, Switzerland; 2 Institute of Bioengineering, École Polytechnique Fédérale de Lausanne, Lausanne, Switzerland; 3 Division of Chronic Inflammation and Cancer, German Cancer Research Center (DKFZ), Heidelberg, Germany; 4 Research Unit Radiation Cytogenetics, Helmholtz Zentrum München Research Center for Environmental Health (GmbH), Neuherberg, Germany; 5 Biomedicine Discovery Institute and Department of Biochemistry and Molecular Biology, Monash University, Melbourne, VIC, 3800 Australia; 6 PhD Program in Systems Biology, Life Science Zurich Graduate School, University of Zurich and ETH Zurich, Zurich, Switzerland; 7 Faculty of Science, University of Zurich, Zurich, Switzerland

**Keywords:** Prognostic markers, Mass spectrometry

## Abstract

To-date, most proteomic studies aimed at discovering tissue-based cancer biomarkers have compared the quantity of selected proteins between case and control groups. However, proteins generally function in association with other proteins to form modules localized in particular subcellular compartments in specialized cell types and tissues. Sub-cellular mislocalization of proteins has in fact been detected as a key feature in a variety of cancer cells. Here, we describe a strategy for tissue-biomarker detection based on a mitochondrial fold enrichment (mtFE) score, which is sensitive to protein abundance changes as well as changes in subcellular distribution between mitochondria and cytosol. The mtFE score integrates protein abundance data from total cellular lysates and mitochondria-enriched fractions, and provides novel information for the classification of cancer samples that is not necessarily apparent from conventional abundance measurements alone. We apply this new strategy to a panel of wild-type and mutant mice with a liver-specific gene deletion of Liver receptor homolog 1 (Lrh-1^hep−/−^), with both lines containing control individuals as well as individuals with liver cancer induced by diethylnitrosamine (DEN). Lrh-1 gene deletion attenuates cancer cell metabolism in hepatocytes through mitochondrial glutamine processing. We show that proteome changes based on mtFE scores outperform protein abundance measurements in discriminating DEN-induced liver cancer from healthy liver tissue, and are uniquely robust against genetic perturbation. We validate the capacity of selected proteins with informative mtFE scores to indicate hepatic malignant changes in two independent mouse models of hepatocellular carcinoma (HCC), thus demonstrating the robustness of this new approach to biomarker research. Overall, the method provides a novel, sensitive approach to cancer biomarker discovery that considers contextual information of tested proteins.

## Introduction

Cancer biomarkers are key to the diagnosis and treatment of disease, and recent developments in proteomics are now permitting a more comprehensive examination of protein biomarkers^1^. Thus far, only a small number of protein biomarker candidates have been translated into the clinic, underlining the limitations and challenges of current approaches to biomarker discovery and identification^2^. These validated protein diagnostics predominantly come from blood plasma or serum samples^3^, yet tissue-based protein biomarkers are particularly valuable for the characterization of tumor tissue types (prognostic markers) and to inform therapeutic decisions (therapeutic markers). In this respect, the emerging field of personalized/precision medicine is highly dependent on tissue-based protein biomarkers, specifically due to the high complexity and heterogeneity of tumors. Typically, protein biomarker discovery projects have significantly relied on quantitative mass spectrometry (MS). The most common approaches to biomarker identification have been the comparison of the abundance of proteins across groups, whether binary case vs. control or continuous population-wide cohorts. However, depending on the biological or clinical state, proteins may not only change their abundance within cells or tissues but also their cellular context, e.g. their subcellular localization or their association with protein complexes. For example, protein intracellular mislocalization has become a key parameter for understanding the development of malignancy and mechanisms for anticancer treatments^4–6^. It has been reported that the increased localization of β-catenin to the nucleus is associated with an increase in oncogenic properties in colon cancer^7^. The subcellular location of proteins has been investigated extensively by antibody-based strategies^8^, also with the aim of detecting context-dependent biomarkers. The proteomes of subcellular compartments have also been systematically investigated by MS-based proteomic techniques^9–13^, in particular following organelle-specific enrichment protocols^14,15^. Such studies are used for localizing individual subcellular proteomes^16^, and have resulted in valuable, generic proteome maps of several subcellular structures, or are used in a conventional way as organelle-enriched tissue extracts for discovery of biomarkers by quantitative comparison control vs. disease state^17^. However, the detection of state-specific changes in proteome distribution across diverse subcellular compartments and in a case vs. control group hasn’t yet been attempted by systematic MS screening.

Here, we hypothesize that the cellular context of proteins, specifically their subcellular distribution, is informative in liver cancer detection. We expect changes in the protein distribution between cytosol and mitochondria to be particularly informative, since mitochondrial proteins play a central role in cancer bioenergetics, survival and proliferation^18^ which are ubiquitous hallmarks of cancer^19^. In this study we tested this hypothesis in a cohort of liver-specific Lrh-1^hep−/−^ mutant mice and their control littermates that were either untreated or had developed a chemically-induced liver cancer after 6 months of DEN treatment. Lrh-1 deletion causes distinctive metabolic perturbations related to hepatic glucose and steroid metabolism^20^ and cellular proliferation, and therefore represents an important factor in cancer development^21,22^. Discovered proteins that altered their mitochondrion concentration (mtFE score) in DEN-treated hepatic tissue of both mice genotypes, either due to its intracellular translocation or due to its variation in expression, were successively verified in independent experiments. These validation experiments involved two distinct mouse models of hepatocellular carcinoma (HCC), which was induced either with 10 months DEN chemical treatment (long-term DEN cohort) or by long-term choline-deficient high fat diet (CD-HFD) for 12 months leading to non-alcoholic steatohepatitis (NASH)-induced HCC^23^. Overall, the study indicates that protein biomarkers identified by the mitochondrial fold enrichment (mtFE) score that we introduce here, outperform biomarkers defined strictly by protein abundance.

## Results

### Study design and MS data acquisition

**Figure 1:**
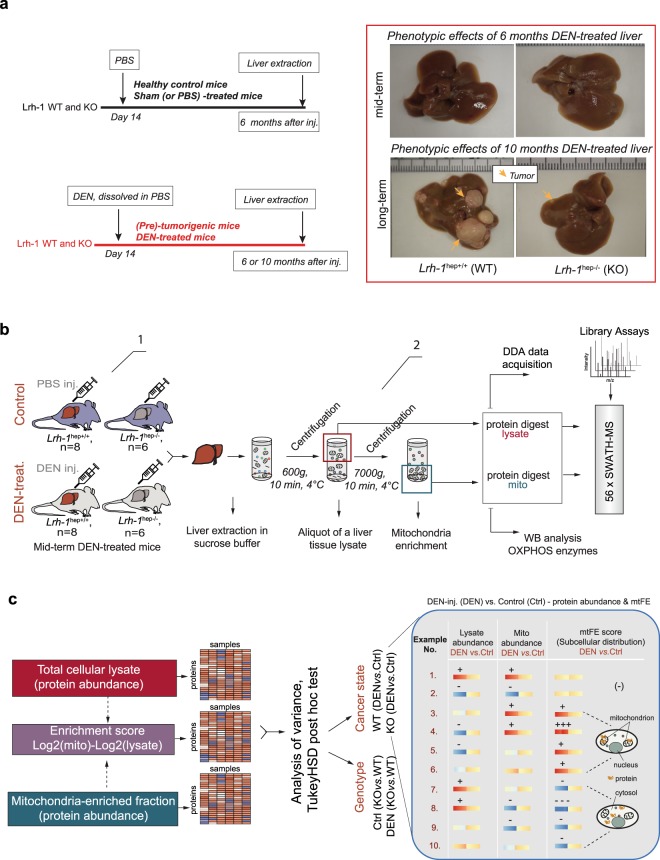
Mouse model and study design used in the discovery experiment. (**a**) Liver tumor induction by DEN administration in Lrh-1^hep+/+^ (WT) and Lrh-1^hep−/−^ (KO) mice. Six or ten months after injection (mid-term DEN and long-term DEN, respectively) mice were sacrificed, and liver tissue collected (left). Phenotypic effect of mid- and long-term DEN-treated liver in the corresponding genotypes (right). Images are representative of pictures obtained from 5–8 mice per genotype. Arrows point to developed tumor nodules particularly expounded in WT long term DEN-treated livers (right, lower panels). Lrh-1 KO mice are less sensitive to DEN-induced carcinogenesis as previously demonstrated on the identical mice cohort^21^. (**b**) Study workflow. From left to right: 1. mid-term DEN-induced tumorigenesis in WT and KO mice in comparison with PBS-treated control groups. *n* corresponds to the number of mice per group in mid-term DEN cohort. 2. Liver homogenization, collection of aliquots for total cellular lysate, and organelle extraction by differential centrifugations steps in a sucrose isolation medium. Parallel SWATH-MS analysis of enriched mitochondrial fraction and total liver lysate. (**c**) Differential analysis of proteomic data generated by SWATH-MS or data indirectly computed from the quantitative proteomic measurements (left). The hypothetical models of “DEN-treated vs. Control comparison” illustrating the effects of different protein abundance levels in total lysate and mitochondria fraction on its subcellular distribution (Example number 1–10, e.g. positive mtFE value indicates protein presence in the enriched fraction).

To examine the hypothesis that subcellular distribution is an informative parameter for protein biomarker detection, we used a mouse model with a liver specific gene deletion of *Liver receptor homolog 1* (Lrh-1^hep−/−^) and their wild-type littermates (Lrh-1^hep+/+^)^21^(Fig. 1a). Lrh-1 (also referred to as Nr5a2) is a nuclear receptor that is abundant in the liver where it has diverse known functions. These include control of cell growth and proliferation and a role in the maintenance of glucose and cholesterol homeostasis^24^. It has recently been shown that Lrh-1 has a pro-tumorigenic activity in hepatocytes by promoting cancer cell metabolism through an alternative glutamine pathway^21^. In this study, we used Lrh-1^hep+/+^ and Lrh-1^hep−/−^ mice in which liver tumors were chemically induced by diethylnitrosamine (DEN) administration at 14 days of age (Fig. 1a, left). While long-term DEN-challenged WT mice (i.e. long-term cohort = 10 months treatment) developed multiple hepatic tumors, Lrh-1-deleted livers presented a significantly smaller tumors and moderate protection against hepatocellular carcinoma development (Fig. 1a, right). In mid-term (i.e. mid-term cohort = 6 months treatment) DEN-treated livers, the tissue morphology of both genotypes did not visibly differ in the presence of early tumorigenic changes (Fig. 1a, right). For our initial discovery experiment, liver tissues were collected from DEN-treated tumorigenic mice of both genotypes at 6.5 months of age and PBS-treated control animals and further processed for MS analysis (Fig. 1b).

To detect changes in protein abundance and changes in the cytosol-to-mitochondria distribution of proteins across the four mouse groups, we extracted proteins from each liver sample from total cell lysate (lysate) as well as from a mitochondria-enriched fraction (mito) (Fig. 1b). All samples were analyzed by quantitative proteomics via SWATH/DIA-MS. Mitochondria enrichment relied on a previously developed protocol^25^. It consists of the relatively fast isolation of a crude mitochondrial pellet (CMP) from mouse liver by a series of differential centrifugations steps in a sucrose isolation medium (Fig. 1b). The protocol is similar to other well-established standard protocols for CMP^15^ and is suitable for parallel processing of high sample numbers without introducing significant variations due to complicated enrichment steps. We processed 28 liver samples of mid-term DEN-cohort from four distinct groups of mice: WT (Lrh-1^hep+/+^), control and DEN-treated tissue (*n* = 8, both groups) and KO (Lrh-1^hep−/−^), control and DEN-treated tissue (*n* = 6, both groups) (Fig. 1b).

SWATH/DIA-MS was used for data acquisition of total tissue lysate or mitochondria-enriched fractions, due to its high degree of reproducibility and quantitative accuracy (Supplementary Fig. 1a)^26–28^. From the acquired spectral maps we detected 2578 and 2558 proteins from total cell lysate (lysate) and mitochondria-enriched fraction (mito), respectively, both at controlled protein FDR of 1% (Supplementary Fig. 1a,b, Tables 1, 2). For this we used a murine liver SWATH library containing 3945 distinct protein groups (28′331 unique peptide sequences) that was generated by using previously described tools^29^ from available data dependent acquisition (DDA) measurements of mitochondria-enriched, nuclear or whole cellular lysate fractions extracted from mouse liver. As expected, most proteins from the mitochondria fraction (i.e. 85.9% (2377 proteins)) were also detectable in total cellular lysate. Overall, we identified 2759 distinct proteins (SwissProt identities), of which we reproducibly quantified 2735 proteins across the mice cohort (Supplementary Table 3) by enabling the requantification feature for all peptide groups identified in more than 5 MS runs^30,31^. In addition to conventional protein abundances measured both in mitochondria-enriched fractions and total cell lysate, we defined and computed for each protein a discriminant score, the mitochondrial fold enrichment (mtFE) score, which indicates the cellular context of quantified proteins. mtFE was calculated as the fold change difference between the log2 abundance values of proteins quantified in mitochondria-enriched fractions and full lysates (i.e. mtFE = Log2(Mito)-Log2(Lysate)) (Fig. 1c). Recent advances in proteomic technology, specifically the development of the SWATH/DIA-MS method, have made this approach feasible due to the highly accurate, reproducible and consistent peptide quantification with minimum interference of sample type/background^28,32^ and the moderately high sample throughput due to single injection per sample. While indirectly derived from abundance values, the meaning and behavior of the mtFE score is fundamentally different from standard protein abundance comparisons between tested conditions (e.g. DEN vs. Ctrl, Fig. 1c). First, the mtFE score is informative for both, the magnitude of the abundance change and the protein cellular distribution (whereby a greater value indicates a significant segregation of protein in the enriched fraction) (right, Fig. 1c, example No 3–6). Second, mtFE is sensitive to relatively moderate protein abundance changes in opposite directions in the total cell lysate and mitochondria occurring due to protein intracellular redistribution. Such subtle changes may not be detectable by conventional whole cell analyses (right, Fig. 1c, example No. 6, 10). Importantly, the implementation of the mtFE score results in the identification of different subsets of significantly regulated proteins that are experiencing changes in both subcellular localization and abundance.

### Evaluation of average mtFE score for organelle marker proteins

**Figure 2:**
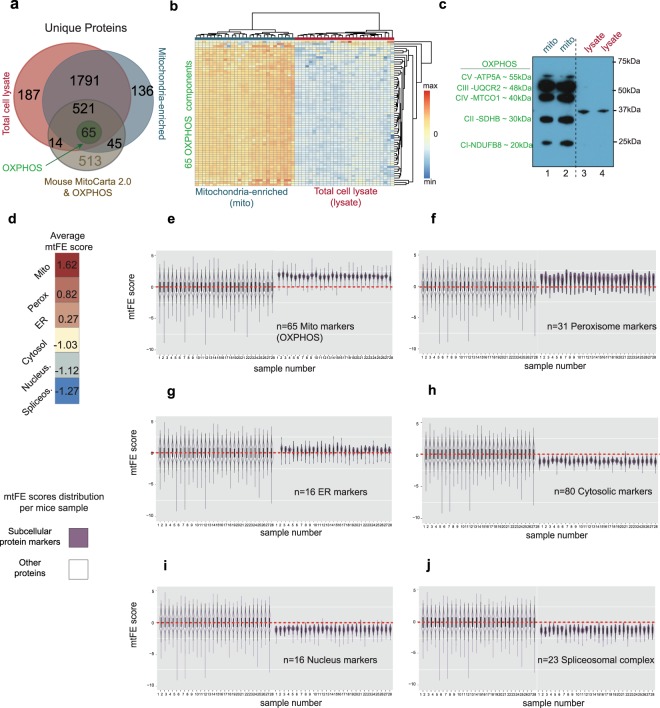
Control of data quality. (**a**) Venn-diagram represents intersection of proteins identified from total lysate and enriched-mitochondrial fraction of the 28 liver samples, with 645 proteins localized in mitochondria according to MitoCarta2.0 mouse inventory. (**b**) Heat-map shows the stronger relative intensity of 65 OXPHOS enzymes in the mitochondria-enriched fractions compared with cellular lysate. (**c**) After very short membrane exposure (t = 5 s) by chemiluminescence, Western Blot of five OXPHOS enzymes visible only in concentrated mitochondrial fraction (lanes 1 and 2) and not in cellular lysate (lanes 3 and 4). Full membrane picture available in Supplementary Fig. 7. A longer exposure in Supplementary Fig. 2a represents gradual appearance of OXPHOS components in cellular lysate. (**d**) The average mtFE score for protein markers of subcellular compartments: mitochondrion, peroxisome, ER, cytosol, nucleus, and spliceosome (**e**–**j**). The violin plots in dark violet present separately the difference in the distributions of mtFE scores of selected protein markers: (**e**) mitochondrion, (**f**) peroxisome, (**g**) ER, (**h**) cytosol, (**i**) nucleus, and (**j**) spliceosome. The violin plots in white color present distributions of all other measured proteins. n corresponds to the number of the respective subcellular markers per individual violin plot in dark violet.

Of the quantified 2735 proteins, 645 protein IDs were associated with “mitochondrion” (Fig. 2a, Supplementary Table 4) according to the Mouse MitoCarta2.0 database^33^. Of these, 65 proteins were components of Oxidative Phosphorylation (OXPHOS) enzymes (Fig. 2a,b) located in the inner mitochondrial membrane. Mitochondria enrichment was carried out successfully in all 28 samples, as confirmed by the MS-measured protein abundance of 65 detected OXPHOS components, which was consistently higher in the mitochondria-enriched fraction compared to the cellular lysate samples even when we injected identical amount of peptides per sample for both preparations (Fig. 2b).

The quantitative proteomic measurements between the total cell lysate and mitochondrial fractions was further confirmed independently by “short exposure” Western Blot detection of five OXPHOS proteins in mitochondria-enriched fractions (lane 1–2) compared to lysate (lane 3–4) (Fig. 2c) and after longer exposure (Supplementary Fig. 2a).

A large fraction of the mitochondrial proteins, including 65 OXPHOS proteins, were consistently identified in the SWATH/DIA-MS data of both, total cellular lysate and mitochondria fraction (Fig. 2a,b), thus allowing for an exhaustive mtFE score calculation for a broad range of proteins. Mitochondria exist in a dynamic network and interact closely with other organelles in the cell, most notably the (endoplasmatic reticulum) ER^34,35^. Therefore, a crude mitochondrial pellet (CMP) always contain a certain amount of impurities from other organelles and specifically from those that are in close proximity to mitochondria inside the cellular environment^15^. We reasoned that, if the mtFE represents a truly sensitive measure of mitochondrial protein repartition, it should produce a somewhat graded score when applied to proteins belonging to such compartments. We therefore evaluated protein markers of mitochondria (mito), peroxisomes, ER, cytosolic enzymes, nucleus and spliceosome complex based on their distance to crude mitochondria-enriched fractions by calculated mtFE scores, and we found that this is indeed the case (Fig. 2d–j). Accordingly, OXPHOS proteins generally displayed elevated mtFE scores across all individual 28 samples (mtFEoxphos = 1.62, Fig. 2e), in line with their predominant or exclusive localization in mitochondria. Peroxisomes are frequently isolated in CMP due to their close interconnectivity with mitochondria inside mammalian cells^36^. Peroxisome and ER markers (n = 31 and n = 16, respectively), as expected, display closer distance to mitochondria-enriched fractions compared to the cytosolic enzymes (n = 80)(Fig. 2d–h), with a lower average enrichment score compared to mitochondrial proteins (mtFEperox. = 0.82, mtFEER. = 0.27, and mtFEcytosol = −1.03 *vs*). The average enrichment score of 64 nuclear markers was still lower than that of cytosolic enzymes (mtFEnucleus = −1.12, Fig. 2i), while 23 members of the spliceosome complex displayed a substantial exclusion from mitochondria and their surrounding environment (mtFEspliceosome = −1.27, Fig. 2j). Overall, these observations suggest that the mtFE score reflects reliably and quantitatively mitochondrial repartition.

Next, we assessed the technical variation of the mtFE approach. We used additional WT mouse liver (i.e. 5 pieces from identical liver tissue), independent of the 28 tested samples, and acquired data from experimental replicates, i.e. five repeated extractions leading to 5 mitochondria-enriched and 5 lysate preparation, respectively. All measured protein abundances including OXPHOS proteins were similarly distributed between replicates of total cellular lysate, mitochondria-enriched or calculated mtFE scores (Supplementary Fig. 2b). The coefficient of variation (CV) of 65 OXPHOS proteins measured among mitochondria-enriched fractions ranged between 1.3% and 22.1% and had a median value of 6% (Supplementary Fig. 2c). Remarkably, calculated mtFE data and total protein lysate data had comparable CV ranges (2.9% to 39.9% and 3.6% to 38.8%, respectively) and median values (10.8% and 10.4%, respectively) (Supplementary Fig. 2c). We performed a correlation analysis of the measurements across all 15 samples, which showed that the five replicates of the three respective data types clustered distinctly and that the similarity within fraction types were high (correlation of ~0.75 to ~0.90) (Supplementary Fig. 2d). As anticipated, weaker or negative correlation was observed between mtFE and the other two datasets (Spearman’s rho range = |0.30|–|0.35|) since mtFE scores do not contain the same information about proteins as conventional expression data. Taken together, these data confirm a high degree of reproducibility of our workflow for generating quantitative proteomic data matrices. Likewise, the calculated mtFE scores that combine the experimental variation of either method—lysate and enriched mitochondrial fraction—also presented a high degree of reproducibility and contain information that is neither contained in the full lysate nor the mitochondria-enriched dataset alone.

### Statistical analysis of three data matrices to evaluate genetic and cancer state differences

**Figure 3:**
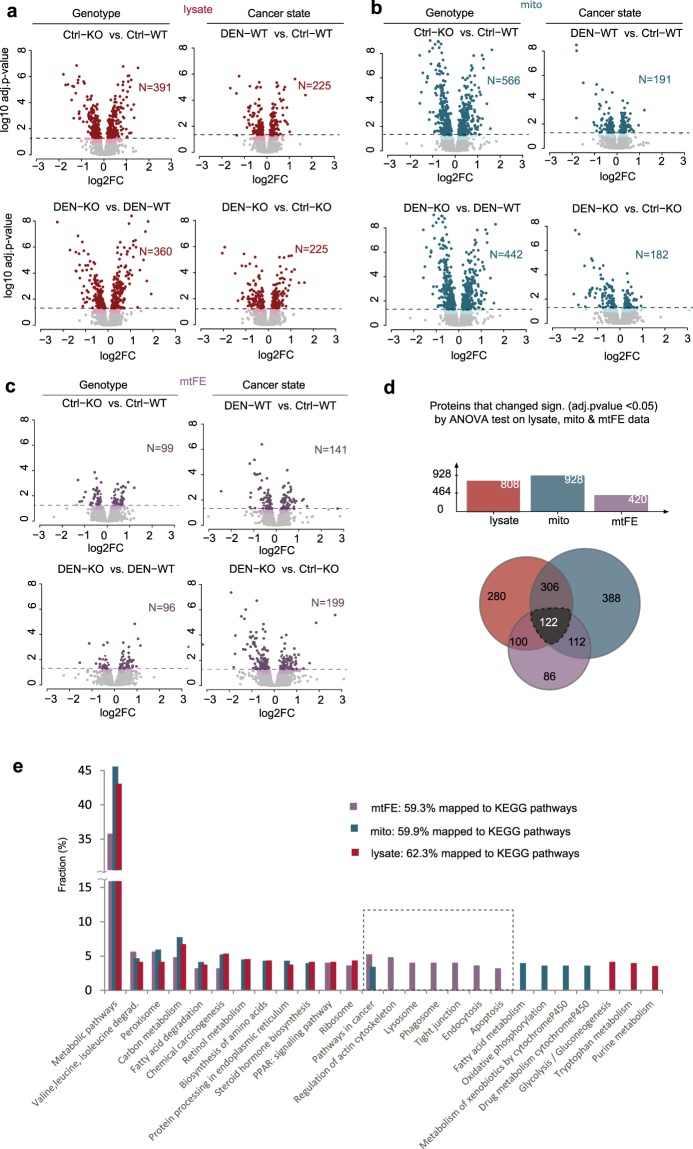
Differential analysis of three data matrices across conditions. Volcano plots corresponding to four comparisons by ANOVA test in (**a**) total cellular lysate (**b**) mitochondria-enriched fractions and (**c**) mtFE data. Differentially expressed proteins (adj. p-value < 0.05) can be seen in dark red (lysate), dark green (mito) or dark violet (mtFE scores). Pastel-colored points correspond to moderate protein changes (adjusted p between 0.05 and 0.1), while light gray points represent unaffected liver proteins. The comparisons between conditions correspond either to differences in genotype or cancer state. The N represents the number of significantly changed proteins (adj. p-value < 0.05) for each comparison. (**d**) Venn diagram results and its summary graph of shared significantly regulated proteins between three datasets (ANOVA test, adj. p-value < 0.05). (**e**) Mapping of KEGG cellular pathway database with significant proteins from the three datasets. Y-axis represents the percentage of total mapped proteins in KEGG database that corresponds to specific cellular pathway on X-axis.

To identify proteins that are differentially regulated across the 4 conditions - control (Ctrl) and DEN-treated livers (DEN) for both genotypes, Lrh-1^hep+/+^ (WT) and Lrh-1^hep−/−^ (KO) - we performed a one-way ANOVA-test on the three protein datasets (total cellular lysate (lysate), mitochondria-enriched fraction (mito), and mtFE score), followed by Tukey’s HSD (honest significant difference) post hoc test to check significant changes. To perform an objective comparison and to maximize the level of information extracted from the three independent data matrices, we considered multiple-testing adjusted p-values of <0.05 rather than a fold change cutoff (FC). We then combined the list of ANOVA-significant proteins for the four distinct comparisons related either to the genotype (KO *vs*. WT, in DEN and Ctrl tissue) or cancer state difference (DEN *vs*. Ctrl, for both genotypes) into a single list (Supplementary Fig. 3a–c). 928 proteins were significantly affected in the mitochondrial dataset, 808 in lysate and 420 in mtFE, consistent with the stringent, localization-sensitive behavior of the mtFE (Supplementary Fig. 3a–c and Table 5). To visually inspect ANOVA based changes detected in the two conventional proteomic data sets, we prepared four volcano plots that correspond to individual comparisons such as the genotype or cancer state comparisons (Fig. 3). We found that cellular lysate protein abundance is particularly sensitive to Lrh-1 genetic background, and less so to the cancer state. The number of significantly up- and down-regulated proteins between the different Lrh-1 genotypes tested exceeded the number of differentially regulated proteins between cancer and basal-liver state by more than 50% in both cohorts (Fig. 3a; left and Supplementary Fig. 3a). We observed a similar trend, i.e. genotypic effects dominating disease state effects, for the mitochondria-enriched fractions, resulting in a disproportionately higher number (>150%) of significantly regulated proteins across different genotypes than cancer and basal states. (Fig. 3b; right, Supplementary Fig. 3a,b). In contrast, we observed that protein changes in mtFE scores are more sensitive to the cancer state than to the genotype difference for both cross-comparisons (Fig. 3c; left). The number of mtFE significant changes, caused by cancer formation is around as twice as many proteins comparing to changes caused by Lrh-1 deletion in the mouse liver (Fig. 3c, left; Supplementary Fig. 3c).

Some significant features were common to all three datasets (n = 122) and some were dataset-specific (Fig. 3d). Strikingly, around 20% (n = 86) of the proteins significantly changed by mtFE (Fig. 3d) did not change their abundances in any of the two conventional expression data, indicating that mtFE uncovers previously inaccessible, contextual information (e.g. protein intracellular trafficking). Likewise, KEGG pathway mapping of significant proteins from three datasets revealed that some of the involved cellular pathways were common to all three datasets (i.e. chemical carcinogenesis; Fig. 3e), while some were exclusively discovered by mtFE, enriched-mitochondria or total cellular lysate data. For example, differential changes reflected by mitochondria-enriched fraction were related to oxidative phosphorylation, which takes place inside the inner mitochondria membrane, or fatty acid metabolism, catabolic processes that generate energy from fatty acids by beta oxidation and the citric acid cycle in the matrix of mitochondria organelle (Fig. 3e). Changes reflected in the glucose metabolism, which mainly occurs in the cytosol, were highlighted by measurements of total lysate cellular proteome (e.g. glycolysis/gluconeogenesis; Fig. 3e). Interestingly, some processes that involve intracellular protein transports or protein shuttle between cytosol and mitochondria were exclusively discovered by mtFE data (i.e. phagosome, apoptosis, endosome; Fig. 3e).

### Unsupervised classification of three data matrices: Cytosol-to-mitochondria protein redistribution (mtFE scores) are sensitive to cancer state

**Figure 4:**
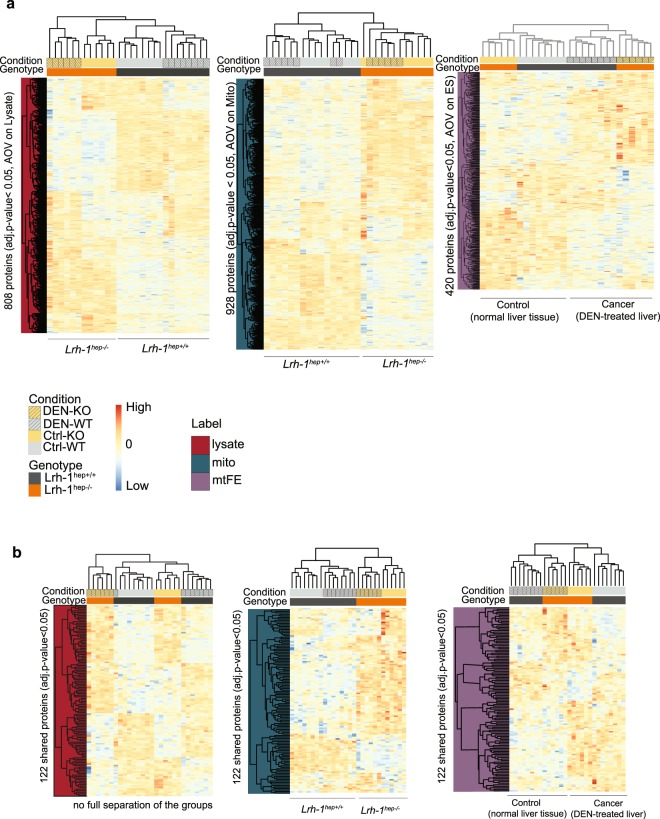
Unsupervised data clustering for three protein data matrices. (**a**) Heat map visualization of data clustering based on subset of significant proteins in lysate data (left panel), mitochondrial data (middle panel), or in mtFE data changes (right panel). Two main data clusters in left and mid panel are divided by genotype differences that correspond to either Lrh-1^hep+/+^ (WT) or Lrh-1^hep−/−^ (KO) livers. (**b**) Clustering based on the subset of 122 significantly changed proteins in all three datasets. Despite the identical input of a relatively small number of entities, mtFE score (right) partitions data primarily based on the cancer state whereas mitochondrial measurements partition primarily based on genotype. Manhattan distances are used for hierarchical clustering.

We tested the capacity of the three datasets to classify cancer and control samples by unsupervised, data-driven methods. Initial unsupervised clustering based on all quantified proteins resulted in different clustering patterns among the three datasets, with no agreement between mouse groups (Supplementary Fig. 4). We then performed unsupervised hierarchical clustering on the expression values of all proteins detected as significantly changed (adj. p < 0.05) for the respective datasets (Mito, *n* = 928; Lysate *n* = 808, mtFE = 420). We found that the abundance data of the mitochondria-enriched and of the total cellular lysate were partitioned primarily based on the liver genotypes, and only secondarily based on the cancer vs non-cancer state, with no complete determination between tested conditions (Fig. 4a; genotype color annotation). Consistent with the results described above, cellular lysate and mitochondria-enriched protein abundance are particularly sensitive to expression difference between genetic backgrounds, and less so to the liver carcinogenesis.

Interestingly, the mtFE score data of 420 significant changes clustered primarily based on the cancer state (Fig. 4a, right, condition color annotation). The patterns generated from the 28 liver tissues based on protein mtFE scores revealed two main clusters driven by presence (or absence) of liver cancer. Each cancer or normal sample’s cluster was in turn divided in two smaller sub-clusters indicating genotypic differences. Whereas mito expression data clusters were able to differentiate the tested groups by genotype, they did not fully discriminate the samples of four examined conditions (Fig. 4a, middle). Consistent with the volcano plot results above, mtFE robustly separated cancer from healthy tissues for both genotypes individually (Fig. 4a, right). To control for a potential clustering bias due to the different, score-specific protein lists used as input, we repeated the same analysis with the 122 proteins that were identified as significant by all three data readouts (Fig. 4b). Surprisingly, this analysis resulted in three markedly different clustering patterns, wherein the lysate expression data did not fully discriminate the comparisons, mito expression data clustered primarily based on liver genotypes and mtFE score data again based on the cancer state (Fig. 4b). Overall, even though the protein input list in these analyses was smaller compared to the analyses discussed above (122 vs 420, respectively), the mtFE remained substantially informative for cancer tissue. Taken together, these classification results indicate that the combination of protein abundance and localization into a single score is capable of discriminating between cancer and non-cancer state even against significant genetic perturbation, and is, in this respect, superior to protein expression data.

### Logistic regression analysis of three data matrices: tissue biomarker prediction in DEN-induced liver carcinogenesis

**Figure 5:**
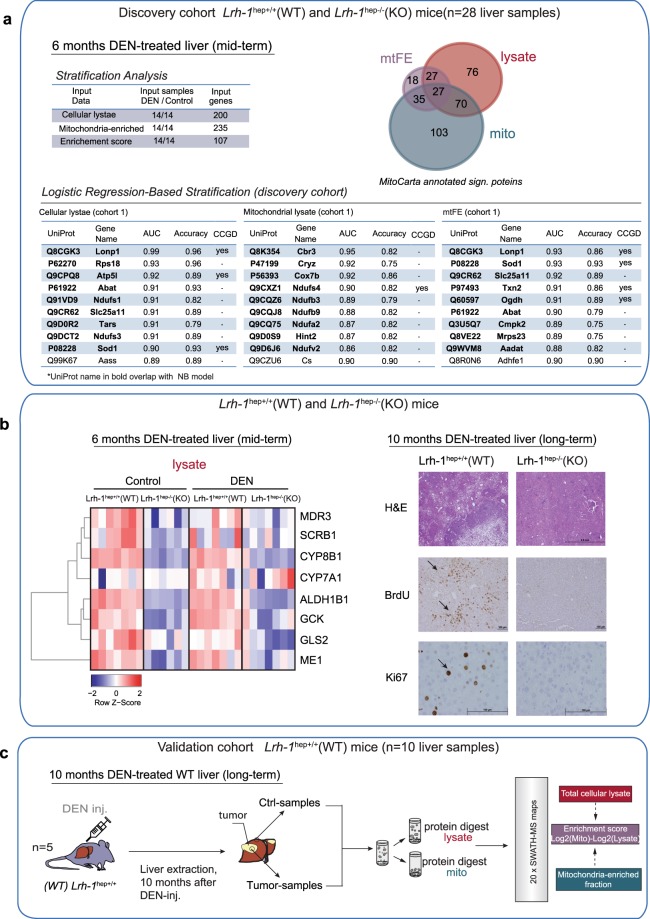
Detection of tissue protein biomarkers in DEN-induced liver cancer. (**a**) Comparisons of three independent data types for DEN-treated liver classification in mid-term mice (discovery cohort/cohort 1). DEN-treated samples from both genotypes (DEN-WT & DEN-KO, n = 14) were grouped and classified against PBS treated controls (Ctrl-WT & Ctrl-KO, n = 14) by logistic regression (LR) analysis. The three input gene lists for stratification analysis include the significant proteins/per data type with evidence of mitochondrion localization. Venn-diagram present intersection of three input lists. Output lists from stratification analysis include top 10 proteins ranked by average AUC values for the three independent analyses (lower panels): lysate (left), mito (middle), or mtFE (right) input data. Protein names highlighted in bold overlap with Naïve Bayes (NB) analysis conducted in parallel on the same data (see also Supplementary Fig. 5a). The CCGD column indicates whether the selected gene is potential cancer driver in hepatic tissue based on information retrieved from the Candidate Cancer Gene Database. (**b**) Confirmation of known Lrh-1 targets in the mid-term (discovery) mice cohort. As expected, conventional expression data present downregulation of several Lrh-1 targets, exemplified by glutaminase 2 (Gls2) and glucokinase (GCK) in KO mice (left). Representative images of hematoxylin-eosin (HE), BrdU and Ki67 immunohistochemical staining of liver sections of 10 months (long-term) DEN-treated Lrh-1hep^+/+^(WT) and Lrh-1^hep−/−^ (KO) mice (right). WT mice present stronger staining of two markers of cellular proliferation, BrdU (5-Bromodeoxycytidine) and Ki67 (MKI67), pointed by arrows (right, lower panels). Images are representative of pictures obtained from 5 mice per genotype. (**c**) We used the long-term DEN-treated WT mice (validation cohort/cohort 2; n corresponds to mice number) to perform the validation of selected candidate markers from the first analysis (i.e., respective panels of discovery cohort in (**a**)). Liver samples (n = 10; 2 paired sample per mouse) collected either from the tumors, or their corresponding surrounding normal tissue from each individual mouse processed and analyzed by identical SWATH/DIA-MS workflow as in the discovery cohort. For each quantified protein in the validation experiment, mtFE score was computed from standard lysate and mito protein data matrices.

We next tested the ability of the three scores generated from the proteomic data of mid-term DEN-treated livers to build predictive signatures for liver cancer in the two genotypes (discovery cohort). To this end, we focused our analysis on those significantly regulated proteins that showed evidence of mitochondrial localization (i.e., proteins annotated in the Mouse MitoCarta Database, Fig. 5a and Supplementary Table 6). To identify the proteins with the highest potential for discrimination between control and mid-term DEN-treated samples (i.e., biomarkers), we used two widely employed machine-learning (ML) algorithms: logistic regression (LR)^37^ and naïve Bayes (NB)^38^ (Fig. 5a and Supplementary Fig. 5a).

For the cancer *(DEN-treated) vs. control* liver tissue stratification, we combined the samples of both available genotypes into single DEN-treated (DEN-WT & KO, *n* = 14) and control group (Ctrl-WT & KO, *n* = 14) (Fig. 5a). For each dataset, we extracted one protein at a time to train and test the ML algorithms via leave-one-out cross-validation. We then ranked and shortlisted the top 10 proteins based on their overall AUC values obtained by LR and NB models (Fig. 5a and Supplementary Fig. 5a, respectively). Of the top 10 proteins obtained via the NB model in the parallel analysis (Supplementary Fig. 5a), as many as 9 overlapped with the LR model for each dataset. A final list of the overlap in the top 10 proteins (i.e., shortlisted by both algorithms) for each dataset and their prediction performance is reported (Fig. 5a, Supplementary Fig. 5a; gene name in bold). Our analysis shows that two sets of top classifiers (i.e. predictive biomarkers to DEN-induced liver carcinogenesis) based on mtFE and lysate data input overlapped for four proteins (i.e., Lonp1, Sod1, Slc25a11, and Abat; Fig. 5a), which further strengthen the statistical and biological credibility of the protein mtFE score. Hence, of the 9 classifiers selected by the lysate data analysis, five proteins, Rps18, Atp5I, Ndufs1, Tars, and Ndufs3, were lysate specific (lysate table, left, Fig. 5a). Notably, the following proteins were unique classifiers based on their mtFE scores: Txn2, Ogdh, Cmpk2, Mrps23, and Aadat (mtFE table, right, Fig. 5a). All classifiers selected by the analysis of the mitochondria-enriched samples were exclusive to this data type showed no overlap with the other two top-protein panels (Fig. 5a, mito table, middle panel). To identify if some of discovered classifiers have been previously identified as a potential cancer driver in mice liver carcinogenesis with relevance to human cancer, we used the Candidate Cancer Gene Database (CCGD)^39^. Remarkably, the highest number of liver cancer drivers was identified by mtFE data classification. Out of 9 classifiers based on mtFE scores, 4 proteins (i.e. Lonp1, Sod1, Txn2 and Ogdh) were identified as potential cancer drivers in a forward genetic screen in mice, in comparison with 1 and 3 in mito and lysate data, respectively (Fig. 5a). The results of the *DEN* vs. *control liver* stratification analysis show that all three data types (lysate, mito, and mtFE) contained unique features with excellent discrimination ability between tumorigenic mid-term DEN-treated samples and their controls (average AUC and accuracy values, respectively; Fig. 5a).

To verify the consistency of the discovered lysate, mito and the mtFE classifiers as effective biomarkers of DEN-induced liver carcinogenesis, we analyzed a new independent cohort of long-term DEN-treated WT mice (validation cohort; Figs 1a, 5b,c). In the long-term study, we extended DEN-chemical injection of mice for a period of four months. Compared to the original mid-term treatment, the long-term study led to development of several macroscopic tumor nodules visible in the morphology of WT liver tissue (Fig. 1a, right lower panel). The DEN-induced liver carcinogenesis was confirmed by the staining of WT liver sections, which demonstrated a strong expression of known markers of cellular proliferation, BrdU and Ki67, comparing to KO (Fig. 5b, right), and the development of several tumor nodules visible in WT liver morphology (Fig. 1a, right, lower panel). We observed that liver carcinogenesis was attenuated in Lrh-1^hep−/−^ KO mice, possibly due to the role of the latter gene in promote carcinogenesis through an alternative glutamine pathway^21^. Our standard lysate proteomics confirms that previously established target genes of Lrh-1 involved in glutamine synthesis, such as glucokinase (GCK) and mitochondrial glutaminase 2 (GLS2)^20,21^, were indeed decreased in Lrh-1-deficient KO livers compared with WT (Fig. 5b, left). Our SWATH/DIA-MS proteomics results were in accordance with initially published analysis of hepatic mRNA and protein (antibody-based method) levels on the identical mice cohorts^21^. Hence, from our validation experiment we excluded Lrh-1^hep−/−^(KO) mice, and concentrated our efforts on long-term DEN-treated WT livers sensitive to DEN-treatment.

**Figure 6:**
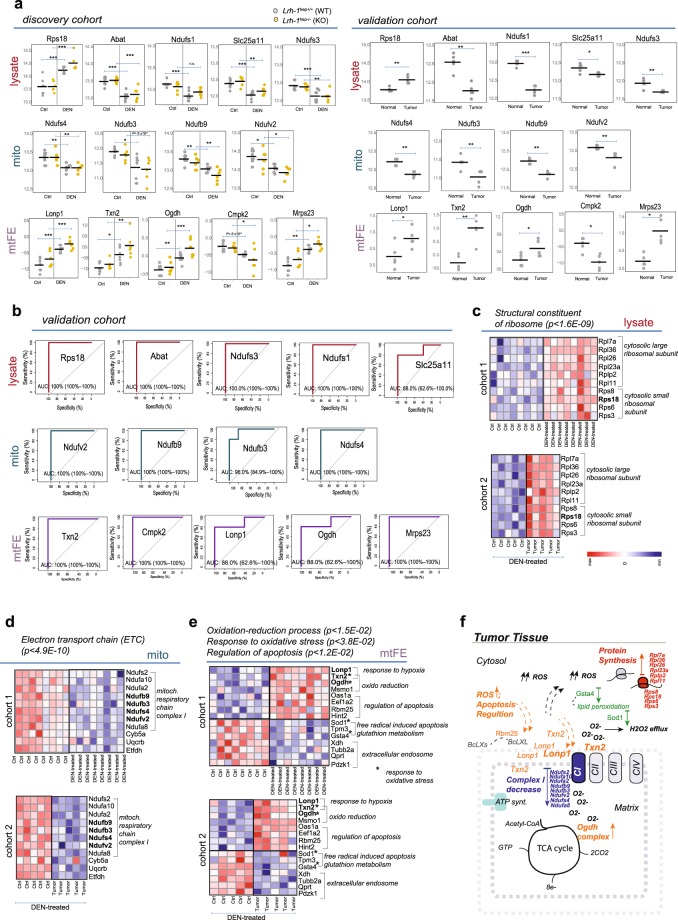
Top protein hits discovered in DEN-induced liver cancer are confirmed in the validation cohort. (**a**) Dot plots show the distributions of the intensities or mtFE values of the tissue markers verified across control and cancer/DEN samples for discovery cohort (left) and validation cohort (right), respectively. P corresponds to either adjusted p-value from ANOVA analysis (discovery cohort) or p-value from independent paired t-test statistics (validation cohort). All dots in gray represent WT mice, while yellow represents KO mice. (**b**) ROC curves computed in the validation cohort represent an estimate of predictive protein ability for three respective data matrices depicted by three color codes: lysate (dark red), mito (dark green) and mtFE (dark violet). (**c–e**) Heat maps for cancer and control samples reflect congruent proteomic changes between the discovery and validation cohorts associated with confirmed biomarkers for lysate (**c**), mito (**d**), and mtFE (**e**) data. P-value of enriched functional categories corresponds to DAVID EASE Score, a modified Fisher Exact P-Value, for gene-enrichment analysis. See also supplementary excel Table S6. (**f**) Graphical representation of intracellular processes revealed by heat maps of lysate (**c**), mito (**d**) and mtFE (**e**) data in surrounding mitochondria environment in tumor tissue. Conventional expression data measured in mitochondria-enriched fraction after extended DEN-treatment, reflects decrease of ETC Complex I located in the inner membrane. Increased ROS further damages Complex I and affects the release of superoxide^60^, while increased accumulation of apoptotic modulators in the mitochondrial inner membrane and for redox-sensitive proteins is visible by depletion of SOD2 mtFE scores, which provokes lipid peroxidation (see marker Gsta4)^61,62^. These processes, reflected exclusively by mtFE markers, can in turn activate the retrograde signaling to cytosol and nucleus^48^ that influence an increase in ribosomal protein expression reflected by total cellular lysate.

We also reasoned that some of the biomarkers discovered in the original study group (mid-term, discovery cohort, Fig. 5a) could be the result of DEN chemical treatment and not liver carcinogenesis, since the control/normal liver samples from both genotypes had been injected with PBS vehicles (Fig. 1a, left). To rule out any distortion of our procedure and results by the confounding effect of DEN treatment, we included in our validation cohort mice treated with DEN only (validation cohort; Fig. 5b, right). From each WT mouse liver, we isolated a tissue section of the tumor, as well as a section corresponding to surrounding normal tissue, 10 months after DEN injection (10 distinct samples; see Fig. 5c). Since both tissue sections (*Ctrl* and *Tumor*) were isolated from the same DEN-treated liver, the confounding effect of the carcinogenic agent should be minimal. SWATH/DIA-MS measurements of mitochondria-enriched and whole lysate fraction were performed, validation samples analyzed and corresponding mtFE scores computed for each protein quantified by standard data matrices (Fig. 5c). Unsupervised clustering based on 2185 quantified proteins (Supplementary Table 7) in this validation cohort reveal a clear discrimination of liver tumor from normal surrounding tissue by all three data (Supplementary Fig. 5b). Of these 2185 proteins, 331 were distinctive to enriched mitochondria fraction. In the same way as the standard liver abundance data, mtFE protein scores of the validation samples remain informative in the tumor tissue characterization. We next applied two-tailed paired t-test statistics to verify differential expression of the above-discovered classifiers in the tumor tissue vs its surrounding normal tissue (9 genes in bold/data type, Fig. 5a) in the validation cohort. Of the 9 above selected candidates (discovery cohort, Fig. 5a) per each conventional abundance-based datasets, 5 proteins confirmed significant changes (paired t-test p < 0.05) in liver tumors for lysate (i.e., Rbs18, Ndufs1, Ndufs3, Abat, and Slc25a11; Fig. 6a, upper panels) and 4 proteins for mitochondria-enriched fraction (i.e., Ndufs4, Ndufb3, Ndufb9, and Ndufv2; Fig. 6a, mid panels). For the mtFE score, of the 9 initial candidates, 5 maintained their significant mtFE score distribution changes (i.e., Txn2, Lonp1,Ogdh, Cmpk2, and Mrps23; Fig. 6a, lower panels). For each protein that remained significant in the validation cohort, we reviewed and plotted the distributions of their intensity values across control and treated samples for each dataset and both cohorts (Fig. 6a), respectively. We generated the ROC curves and presented AUC values on the independent validation samples of the confirmed lysate, mito, and mtFE score-based regulated proteins (Fig. 6b). Similar to the markers of total lysate and mitochondria-enriched fraction, discovered mtFE changes correctly stratified tumor tissue in the samples independent of the original ML analysis. Summary statistics of ROC analysis was computed on the validation cohort for confirmed markers of three data matrices for DEN-induced carcinogenesis (Supplementary Fig. 5c). Maximal AUC values of three selected markers, Tnx2, Cmpk2, and Mrps23 (AUC = 100%) and its relatively high values for two proteins, Ogdh and Lonp1 (AUC = 88%), confirm the consistency of protein mtFE scores in the stratification of hepatic tumor (Fig. 6b). Our validation results clearly demonstrate that mtFE scores were indeed as effective as using protein abundance values to discriminate between DEN-induced cancer and normal liver tissue state (Fig. 6a,b).

Additionally to verification analysis of selected classifiers, we perform differential analysis between tumor and their corresponding normal tissue sections (paired t-test, p < 0.05) for proteins quantified in the validation and compare the results with discovery analysis. Besides validated top markers, out of 2093 proteins common quantified in discovery and validation cohort (Supplementary Fig. 5d), 73 remain significantly regulated between both cohorts for lysate, 76 for mito, and 27 for protein mtFE scores data, respectively.

### Biological insights into DEN-induced liver carcinogenesis

The changes in protein expression or enrichment scores caused by the DEN-induction of hepatic cancer were further examined for functional relationships. Specifically, we asked whether we could combine the information obtained by the three orthogonal scores to formulate a coherent biological interpretation for DEN-induced hepatic carcinogenesis. The list of significantly regulated proteins common in both DEN-treated mice groups (Supplementary Fig. 5d, right) were examined for the most prominent biological functions associated with confirmed biomarkers for lysate, mito and mtFE data (Fig. 6a,b). To annotate and visualize metabolic changes of neoplastic liver tissue reflected by the three data types, we used available DAVID Bioinformatic Resources v.6.8^40^ and literature search for single biomarkers (Fig. 6c–e).

We found that even though we could not detect any overlap between validated protein classifiers of two conventional proteome dataset, similar cellular processes were reflected by functional categories of regulated proteins (Supplementary Table S8). In the total lysate we observed significant enrichment of ribosomal proteins and increased tumor abundance of several structural ribosomal subunits: 6 proteins of the large ribosomal subunit, and 4 of small ribosomal subunit including top classifier Rps18 (Fig. 6c). Alongside the ribosomal proteins as the most significant functional category, decreased abundance of markers of electron transport chain (ETC) respiratory complex I was also reflected by liver lysate as exemplified with two markers Ndufs1 and Ndufs3 **(**Fig. 6a) and the second annotation cluster in the functional analysis (Supplementary Table S8). This is in accordance with mitochondria-enriched data, where among regulated proteins, several enriched subunits of ETC complex I showed the main functional category and decreased abundance in the tumor samples (Fig. 6d), and four of them are, in fact, identified as best performing markers of tumor tissue for this mito dataset (i.e., Ndufs4, Ndufb3, Ndufb9, and Ndufv2; Fig. 6a).

By contrast, mtFE data analysis, suggested distribution changes of key proteins - Txn2, Lonp1 and Ogdh -related to the cellular redox system and regulation of apoptosis/cell survival in respect to mitochondrial fraction (Supplementary Table S8, see also KEGG-based pathway analysis, Fig. 4b). Due to the smaller number of confirmed regulated features for mtFE data (i.e. 27 proteins, Supplementary Fig. S5d), which can reduce stringency of the functional annotation clustering for the most relevant categories, the top mtFE classifiers were verified by a survey of the relevant literature. For instance, Lonp1 and Txn2 are frequently induced by various stimuli in the cell, including hypoxia and the production of reactive oxygen species (ROS), and can therefore regulate cancer cell apoptotic resistance^41,42^. Lonp1 is a major regulator of mitochondrial homeostasis, for which downregulation in cancer cells is known to induces massive caspase 3 activation and apoptotic death^43^. Txn2, a mitochondrial redox-sensitive protein, is a key player in the regulation of mitochondria-dependent apoptosis induced by ROS production^44^. Besides ETC Complex I (i.e. enriched by significant changes in conventional mito data) as the major ROS source, several other sites in the mitochondria could produce ROS. Interestingly, Ogdh, one of the five top mtFE classifiers (Fig. 6a), is a subunit of the OGD complex in the mitochondrial matrix, which significantly contributes to ROS production^45^. Other dysregulated mtFE protein scores in DEN-induced tumor tissue also illustrate apoptotic dysregulation as indicated by increased accumulation of an apoptotic regulator with known nuclear localization in the liver mitochondrial fraction, RNA binding motif protein 25 (Rbm25). Interestingly Rbm25 exerts function as splicing factor by regulating the balance of pro- and anti-apoptotic transcripts of the gene Bcl2l1^46^.

The corresponding heat maps of the key dysregulated functional categories in tumorigenesis for three proteome dataset—lysate, mito and mtES scores—show consistent abundance trend or mtFE score patterns between cancerous and non-cancerous livers in both mice DEN-cohorts (Fig. 6c–e). Most important, the observed mtFE scores confirmed several key metabolic processes related to mitochondria in tumor tissue that would not be discovered neither by regular quantitative proteomic measurements nor direct measurement of single mitochondria liver fraction.

We then established a model of hepatic tumor formation induced by DEN treatment that combines the biological insights gained from protein mtFE readouts with the complementary information obtained from standard proteomic data, (Fig. 6f). DEN-induced liver carcinogenesis presents a severe well-known toxic impact on mitochondrial respiratory chain activity by increased production of highly reactive oxidative molecules^47^, as suggested by the striking abundance decrease in ETC Complex I and mtFE score increase of mitochondrial enzyme Ogdh in tumor tissue (Fig. 6d–e). Increased ROS production leads to a respiration rate decrease by oxidative damage, low ATP production, and hypoxia^48^, which, in turn, activates Lonp1 and Txn2 mediated cellular resistance to apoptosis by their accumulation in the mitochondrion (mtFE scores; Fig. 6e). Oxidative damage, accompanied by the initiation of antioxidant protective mechanisms (e.g. Sod, Gsta4 mtFE scores heatmap, Fig. 6e) can provoke retrograde redox signaling from the organelle to the cytosol and nucleus, and reprogram cellular metabolism to maximize macromolecule biosynthesis for cancer growth and proliferation^48^. Finally, increased expression of ribosomal proteins (lysate data, Fig. 6c), the basal cellular machinery involved in protein synthesis, could be related to intensive cell proliferation and tumor growth in mice liver. Hence, DEN-hepatic tumor formation induces cellular pathways that are known as hallmarks of cancer such as apoptotic escape, proliferative signaling, response to hypoxia and dysregulated cellular energetics. These tumor tissue changes were better characterized only by connection of three different proteome level data, and from this observation, we conclude that the three proteome analyses are valuable sources of metabolic information, and that their results are more complementary than mutually exclusive.

### Lonp1 and Ogdh protein mtFE scores, validated classifiers of DEN-induced liver carcinogenesis, remain significantly dysregulated in NASH-induced model of HCC

Finally, we decided to challenge our panel of mtFE markers with a different model of HCC, triggered by a distinct aetiology (fatty liver disease), to verify whether they would remain reliable indicators of hepatic carcinogenesis, entirely independent of DEN treatment^23^. An additional important difference to the DEN-induced model is the independence of a direct pro-carcinogenic trigger.

**Figure 7:**
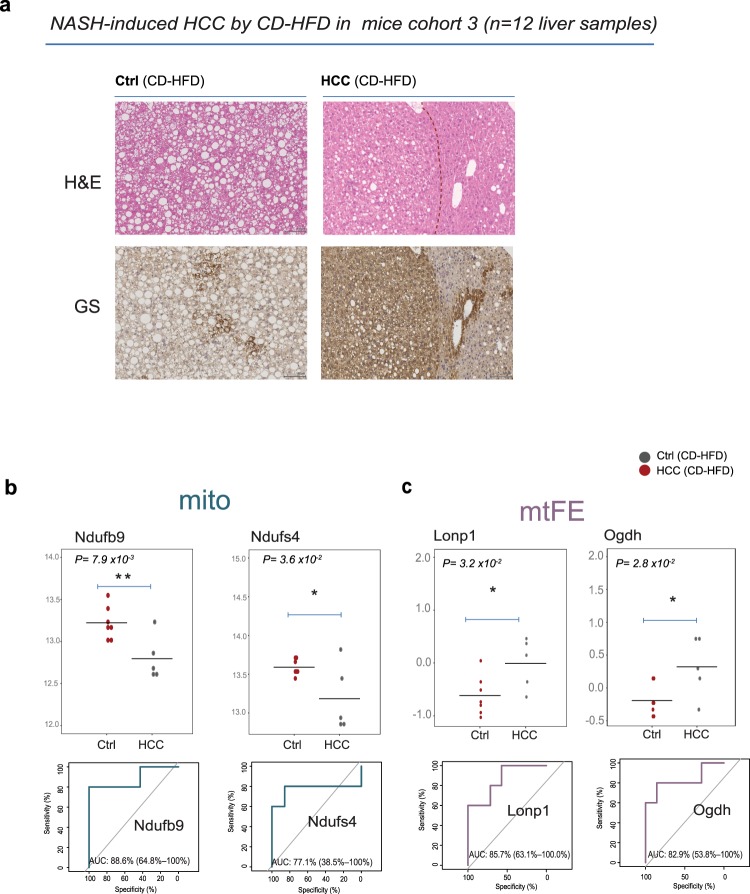
Performance of the individual markers of DEN-carcinogenesis in the NASH-induced model of HCC. (**a**) Representative pictures of hematoxylin-eosin (HE) and Glutamine Synthetase (GS) immunohistochemical staining of liver sections in CD-HFD mice cohort (cohort 3). HCC livers present stronger staining of GS that is marker of carcinogenesis, compared to control liver (right, lower panels). A red dashed line for HE distinguish the tumor nodule. Images (scale bar: 100 µm in magnification 10x) are representative of pictures obtained from 5–7 mice per genotype. (**b–c**) Dot plots show the distributions of the protein intensities in two makers (Ndufb9 and Ndufs4 in (**b**)) of mito data and two markers based on the mtFE values (Lonp1 and Ogdh in (**c**)), that were confirmed in NASH-induced model of HCC. P corresponds to p-value from two-tailed Student’s t-test, with assumed equal variances. All dots in dark gray represent HCC, while dark red represents control samples. ROC curves computed in the CD-HFD mice cohort 3 represent an estimate of predictive ability for two mitochondria-enriched markers (**b**) and for two mtFE scores (**c**).

The new cohort consisted of 12 four-week-old mice that were fed a choline deficient high-fat diet (CD-HFD; 45% of calories, Research Diets; D05010402) to spontaneously induce HCC in the context of non-alcoholic steatohepatitis (NASH). Mice were sacrificed at 13 months of age after 12 months upon CD-HFD, and five of them developed diet induced HCC. The other seven mice challenged by the same conditions are used as control samples. Those samples also developed liver steatosis and NASH after long term CD-HFD (Hematoxylin-eosin (HE), Fig. 7a). The representative liver pictures of HE and Glutamine Synthetase immunohistochemical staining demonstrated the presence of heterogeneous cellular composition in five HCC mice that differ from normal liver with physiological cytoplasm/nucleus ratio and with no sign of malignant transformation (Fig. 7a). The pictures of marker of carcinogenesis, Glutamine Synthetase (GS), present significantly stronger staining in the livers that developed HCC compared to controls.

We extracted and measured liver proteomes of CD-HFD mice exactly in the same way as in the previous experiments. The analysis of new samples resulted in the quantification of 2753 proteins (Supplementary Table S9) over two liver fractions, total lysate (i.e 2298) and mitochondria-enriched (i.e. 2374). The initial data clustering based on quantified proteins in three dataset did not clearly distinguished HCC tissue (Supplementary Fig. S6a). Next, we verified on the new set of 12 mice (Ctrl = 7, HCC = 5), previously validated markers of three data types (i.e. 4–5 proteins per lysate, mito and mtFE scores, Fig. 6a,b) with the best prediction to DEN induced liver cancer (i.e. mid- and long- term DEN-treated mice cohorts, Fig. 6a). On these entirely independent mouse model of liver cancer, our analysis (two-tailed Student’s t-Test, p < 0.05) demonstrated that no single of five lysate markers was confirmed as differentially expressed in HCC liver (Supplementary Fig. S6b). In contrast, two of four markers of mitochondria-enriched fraction, Ndufb9 and Ndufs4, remain significantly downregulated and predictive to HCC tissue (Fig. 7b) while other two related to ETC complex present strong tendency of downregulation that did not reach statistical significance in differential analysis (Supplementary Fig. S6c). Remarkably, two of five classifiers based on mtFE scores readouts, Lonp1 and Ogdh, remain significantly increased and predictive to HCC changes (Fig. 7c, Supplementary Fig. S6d). while Mrps23 score present prediction only slightly better than random assignment (AUC = 0.57). Summary statistics calculated for markers that were validated in CD-HFD mice cohort 3, showed Lonp1 and Ogdh mtFE protein scores to be strongly predictive to NASH-induced mouse model of liver cancer (Supplementary Fig. 6e). Our data demonstrated that decreased abundance of two components of mitochondrial respiratory chain complex I remains a stable metabolic marker in the three examined cohorts, representative of two different models of liver cancers. Most important, our results confirmed that two protein markers Lonp1 and Ogdh based on mtFE readouts also remain significantly dysregulated and predictive in the classification of hepatic cancer tissue. While Ogdh enzyme, is structurally and functionally related to ETC complex I situated in the mitochondria membrane and involved in ROS production, Lonp1 is involved in apoptotic escape of malignant cell in elevated cellular ROS levels. Surprisingly, both confirmed mtFE markers as well as one mito marker (i.e. Ndufs4) were annotated by CCGD database as potential cancer drivers of HCC in mice. This clearly confirms the capability of mtFE values to detect accurate protein features in malignant tissue and demonstrate their change in protein intracellular distribution as a key parameter in cancerous cell. Overall, our data monitored near to 3000 unique liver proteins (i.e. 4054 distinct protein groups) among three separate experiments corresponding to three distinct mouse cohorts of cancer (Supplementary Fig. 6f) suggests that protein flux between cytosol and mitochondria could be the stable quantitative score able to demonstrate the common metabolic changes in liver carcinogenesis of different origins.

## Discussion

In this study, we tested the hypothesis that information that considers the proteins in their subcellular context, in addition to their overall abundance measurements, can provide new, valuable biomarker signatures. This hypothesis is based on the notion that proteins, as a class of biomolecules, uniquely display contextual information that is directly reflective of their biochemical role in the cell. Changes in the context of cellular proteins, therefore, display functional changes of the cell that are likely inaccessible by quantitative protein (or transcript) profiling alone. As an example of contextual information we used the re-distribution of proteins between cytoplasm and mitochondria. The principle demonstrated in this study case can easily be extended to other types of contextual information, including redistribution of proteins between different subcellular fractions and between alterations in the stoichiometry of protein complexes^27^. Mitochondria are a preferred subcellular compartment for cancer biomarker studies due to the role of mitochondrial protein complexes in cancer bioenergetics^49^, survival, and proliferation^18^. We therefore propose a new approach to biomarker discovery, centered around the combination of abundance and contextual information, in the present case of protein localization to the mitochondria, and a new discriminant metric, the mitochondrial fold enrichment score (mtFE). To test our hypothesis, we have used a well-established liver cancer model (DEN-induced liver carcinogenesis) in mice with a liver-specific Lrh-1 deletion and their WT littermates, both challenged by DEN-treatment for 6 months (mid-term study). Lrh-1 gene has a pro-tumorigenic activity in liver through promoting cancer cell metabolism via an alternative glutamine pathway^21^. Therefore, this initial mouse model of liver cancer was deliberately chosen to include genetic variability as confounding component when testing a new approach to cancer biomarker discovery. In our discovery experiment, we challenged the organelle enrichment score values to standard proteome matrices based on: 1. differential analysis between four mice groups related either to the genotype (KO vs. WT, in DEN and Ctrl tissue) or cancer state difference (DEN vs. Ctrl, for both genotypes), 2. unsupervised data clustering according to the samples similarity and 3. stratification analysis based on ML approaches typically used for biomarker discovery in human cohorts. The results of differential analysis reported a higher number of significant changes in genotypes tissue comparisons based on conventional protein abundance data, while cancer vs normal tissue comparisons depicted more changes in mtFE scores data (Fig. 3). Evaluation of unsupervised clustering for conventional abundance data (i.e. mito and lysate) consistently resulted in a primary samples clustering based on genetic background, and only secondarily on cancer state (Fig. 4). In contrast, we observed that subcellular localization, combined with protein abundance measurement, i.e. the mtFE score, clearly discriminated normal from cancer samples (Fig. 4). In keeping with these results, we found that selected biomarkers based on stratification analysis of mtFE values as input data could discriminate with high specificity between cancer and non-cancer state with the same efficacy as protein abundance values (Fig. 5a). To validate these results as valuable markers of DEN-induced carcinogenesis, we used a long term DEN-treated WT mice with advanced tumor stage where the control samples are directly selected from tumor surrounding normal tissue in order to eliminate the effect of DEN-treatment. Of 9 markers based on mtFE values identified in the discovery cohort, as many as five were reproducible and highly accurate in distinguishing the tumor from their normal surrounding liver tissue.

To introduce more confounding effects and challenge five validated mtFE markers with other conventional protein abundance markers validated in DEN-induced carcinogenesis, we collected a third cohort of samples with entirely distinct mouse model of HCC induced by NASH upon 12 months of CD-HFD. We tested if some of our markers would remain robust in the classification of the NASH-induced carcinogenesis. Notably, while any of the five previously validated lysate markers of DEN-induced cancer were not successful in discrimination of HCC from control tissue, two markers from mitochondria-enriched data (i.e. Ndufb9 and Ndufs4) and two based on mtFE score values (i.e. Lonp1 and Ogdh) confirmed their differential expression and high prediction power to cancer samples (e.g. AUCs Lonp1 and Ogdh mtFE scores > 0.8, Fig. 7b,c).

An important limitation of this study was the lack of human samples for the evaluation of the performance of the context biomarkers based on organelle enrichment scores. The human tissue could be more heterogeneous and complex for biomarker detection than well-controlled mouse models. For this reason, the evaluation of a new class of context-based biomarkers in humans would be an important follow-up work. In addition, direct application to human clinical samples will still need some development due to smaller amount of human tissue available for isolation of subcellular compartments such as mitochondria.

It is of note that modifying the disease-related subcellular mislocalization of proteins was already acknowledged as an attractive target for pharmacological intervention^5^. In this respect, context-based biomarkers could be valuable to discover relevant intracellular biochemical processes not associated with changes in protein abundance. This is well illustrated in our model of DEN-induced liver carcinogenesis. By leveraging our analysis of protein cellular distribution between cytosol and mitochondria (mtFE protein scores), we could capture several cancer hallmarks, especially pathways linked to apoptotic resistance and oxidative stress. This is indeed not surprising, since mitochondrial organelles play a fundamental role in cell death by apoptosis or necrosis, and therefore biochemical mitochondrial changes are particularly informative for cancer diagnostics and pharmacology^50,51^.

Importantly, the combined data from the discovery and validation cohorts strongly suggested that mtFE-based biomarkers, if not superior to protein expression data, provide profoundly different insights into the biology of hepatic carcinogenesis as compared to abundance values, as well as a complementary set of biomarkers.

In summary, we introduce a new method, dubbed mtFE, which is more sensitive to cancer-induced intracellular changes than traditional biomarkers based on protein abundance and provides complementary information about the underlying molecular processes. mtFE scores do not require additional cost, and can be computationally extracted from the abundance data. This promising approach is bound to become ever more powerful with the improvements of subcellular fractionation methods and quantitative data acquisition strategies, and could potentially accelerate the discovery of new and more reliable biomarkers for a personalized/precision medicine needs.

## Methods

### Mice generation

#### DEN-induced model of liver cancer

Hepatocyte-specific LRH-1 knockout (Lrh-1^hep−/−^) and wild-type (Lrh-1^hep+/+^) mice were previously generated^20^. Congenic neonatal mice were intraperitoneally injected with diethylnitrosamine (DEN, dissolved in PBS) at 14 days of age with a dose of 25 mg/kg body weight to initiate tumor formation^21^. For the discovery experiment, mice were sacrificed six months after DEN-injection (mid-term), and liver tissues were collected and analyzed. In the validation cohort, the animals were sacrificed ten months after DEN injection and liver tissue collected for the analysis. Control mice of both cohorts, mid/term and long term, were intraperitoneally injected with PBS and collected at the same time as respective DEN-induced mice. All animal experiments were performed in accordance with the institutional guidelines and approved by the Swiss authorities (Canton of Vaud, animal protocol IDs 2375 and 2768).

#### NASH-induced model of liver cancer

C57BL/6JOlaHsd mice (n = 12) were purchased from ENVIGO. Four-week-old male mice were fed with choline-deficient high fat diet (Research Diets; D05010402) for 12 months period^23^. After 12 months, mice were sacrificed and livers were verified microscopically by histopathological examination for signs of HCC liver, or small nodules disseminated in the liver. Five mice spontaneously developed HCC, while seven mice challenged with the same conditions were used as control littermates.

### Tissue processing and organelle enrichment

Preliminary experiments have shown that a minimum of 15 mg of liver tissue is required for the reproducible mitochondrial fraction protocol. The fresh liver tissues were collected, and stored frozen until used. Hence, 20–40 mg of frozen liver tissue per mice sample was homogenized in 500 µL sucrose buffer (200 mM sucrose, 20 mM Tris/Mops, 0.2 mM EGTA, and Roche protease inhibitor cocktail, pH = 7.4). 100 µL of homogenized liver was used for digestion of total cellular lysate. The rest of the tissue homogenate was used to enrich mitochondria by using a differential centrifugation steps in the sucrose density solution (200 mM) as described earlier^25^. In brief, homogenate was diluted by sucrose buffer to a volume of 1 mL and centrifuged 10 min at 600 g to pellet cellular debris and heavy organelles. Then, the supernatant was transferred into new tube and diluted by adding 1 mL of sucrose buffer. Finally, crude mitochondrial fraction was sedimented and washed two times by centrifugation for 10 minutes at 7000 *g*. Protein pellets were dissolved in 8 M urea buffer by vigorous vortexing. An equal amount of 100 µg of total protein content was used for digestion of both total cellular lysate and mitochondrion-enriched fraction. Prior to digestion all samples were adjusted to 1 M urea by dilution with 50 mM of ammonium bicarbonate buffer. Overnight digestion was performed with a ratio of 1 µg trypsin for 20 µg protein. Peptide digest was cleaned on MACROSpin Plate-Vydac Silica C18 (Nest Group Inc., Southborough, MA), solubilized in 100 μL of 0.1% aqueous formic acid (FA) with 2% acetonitrile (ACN) and were used for final MS analysis. Indexed retention time (iRT) peptides were added (RT-kit WR, Biognosys) in equal 1 pmol/μL amount into each samples prior to MS injection.

### Western blot analysis

One-dimensional polyacrylamide gel electrophoresis (1D-PAGE) was performed by using precast NuPAGE Novex 4–12% Bis–Tris gels (Invitrogen, Switzerland). 15 µg of protein were loaded onto each well for both the mitochondrial fraction and total lysate. For western blot analysis, the proteins were blotted onto a polyvinylidene fluoride (PVDF) membrane (iBlot Dry Blotting System, Invitrogen), membrane was then blocked with 5% (w/v) non-fat dry milk in TBST buffer (tris-buffered saline, 0.1% Tween 20) for 1 h at room temperature and incubated with Total OXPHOS Rodent WB Antibody Cocktail (#ab110413, Abcam, UK) in diluted concentration of 1:1000 at 4 °C overnight. This antibody cocktail contains five mouse monoclonal antibodies, one each against CI subunit NDUFB8 (#ab110242, Anti-NDUFB8 antibody), CII-30 KDa (#ab14714, Anti-SDHB antibody), CIII-Core protein 2 (#ab14745, Anti-UQCRC2 antibody), CIV subunit I (#ab14705, Anti-MTCO1 antibody), and CV alpha subunit (#ab14748, Anti-ATP5A antibody), all from Abcam, UK. Next day, the membrane was washed four times for 15 min with TBST buffer and subsequently incubated with a secondary antibody, Amersham ECL Mouse IgG, HRP-linked whole Ab (#NA931-100UL, GE Healthcare) at dilutions 1:20,000. The final Western blot signals were developed with an Amersham ECL Prime Western Blotting Detection Reagent (GE Healthcare), by chemiluminescence using darkroom development techniques.

### Generation of mouse liver specific SWATH assay library

Data-dependent acquisition (DDA) for library generation was performed on a TripleTOF 5600 mass spectrometer equipped with a NanoSpray III source and heated interface (AB Sciex, Concord, Ontario, Canada). 55 distinct injections coming from either enriched-mitochondria fraction, enriched nuclear liver fraction or total cellular digest samples were injected onto a C18 nanocolumn packed in-house directly in a fused silica PicoTip emitter (New Objective, Woburn, MA, USA) with 3-μm 200Å Magic C18 AQ resin (Michrom BioResources, Auburn, CA, USA). Reverse phase peptide separation was performed on a NanoLC-Ultra 2D Plus system (Eksigent–AB Sciex, Dublin, CA, USA). The nanoLC gradient was linear from 2 to 35% B (0.1% formic acid in ACN) over 120 min at a flow rate of 300 nl/min and an oven temperature of 70 °C. The nano-LC and MS instruments were operated by Analyst TF 1.5.1 software (AB Sciex). Electrospray ionization was performed in positive polarity at a voltage of 2.6 kV and was assisted pneumatically by nitrogen (20 psi). Tandem mass spectra (MS/MS) were recorded in “high-sensitivity” mode over a mass/charge (*m/z*) range of 50 to 2000 with a resolving power of 30,000 (full width at half maximum [FWHM]). MS/MS spectra acquisition was triggered by DDA mode consisting in a survey scan of 250 ms followed by 20 MS/MS-dependent acquisitions of 50 ms each and generated by collision-induced dissociation (nitrogen) with dynamic collision energy (i.e., rolling collision energy). DDA selection of the precursor ions was as follows: the 20 most intense ions (threshold of 50 counts), charge state from 2 to 5, isotope exclusion of 4 Da, and precursor dynamic exclusion of 8 s leading to a maximum total MS duty cycle of 1.15 s. External mass calibration was performed by injecting a 100-fmol solution of  β-galactosidase tryptic digest. Raw data files (.wiff) were centroided and converted into mzXML as a final format by using openMS. The converted data files were searched using the search engines X! TANDEM Jackhammer TPP (2013.06.15.1 - LabKey, Insilicos, ISB), and Comet (version “2016.01 rev. 3”) against the ex sp 10090.fasta database (reviewed canonical Swiss-Prot mouse proteome database, released 2016.11.01) appended with common contaminants and reversed sequence decoys^52^ and iRT peptides sequence. The search parameters included trypsin digestion and allowing 2 missed cleavages. Included were ‘Carbamidomethyl (C)’ as static and ‘Oxidation (M)’ as variable modifications. The mass tolerances were set to 50 ppm for precursor-ions and 0.1 Da for fragment-ions. The identified peptides were processed and analyzed through the Trans-Proteomic Pipeline (TPP v4.7 POLAR VORTEX rev 0,Build 201403121010) using PeptideProphet^53^, iProphet^54^ and ProteinProphet scoring. Spectral counts and peptides for ProteinProphet were filtered at FDR of 0.01112 mayu-protFDR (=0.947777 iprob). The raw spectral libraries were generated from all valid peptide spectrum matches and converted to TraML format using the OpenMS tool ConvertTSVToTraML (version 1.10.0). Decoy transition groups were generated based on shuffled sequences by the OpenMS tool OpenSwathDecoyGenerator (version 1.10.0) and appended to the final SWATH library in TraML 4 format. Finally, we configured the murine liver spectral library containing high quality MS assays for tryptic peptides from mouse liver proteins (protein groups). The MS assays, constructed from the top six most intense transitions with Q1 range from 350 to 2000 *m/z* excluding the precursor SWATH window, were used for targeted data analysis of SWATH maps.

### SWATH measurement and analysis

All 55 available mice livers (i.e. three mice cohorts (n = 50) and replicates (n = 5)) that corresponds to 110 distinct sample (mito and lysate fraction/liver) were measured on TripleTOF 5600 mass spectrometer operated in SWATH mode as described earlier^32^. Quadrupole settings in acquisition method were optimized for the selection of 64 variable width precursor ion selection windows from 400 to 1200 *m/z* (Supplementary Table 10). Reverse phase peptide separation was performed with a linear nanoLC gradient from 2 to 35% of buffer B (0.1% formic acid in ACN) over 60 min at a flow rate of 300 nl/min and an oven temperature of 70 °C. An accumulation time of 50 ms was used for 64 fragment-ion scans operating in high-sensitivity mode. In the beginning of each SWATH-MS cycle, a TOF MS scan (precursor scan) was also acquired for 250 ms, at high resolution mode, resulting in a total cycle time of 3.45 s. The swaths were overlapping by 1 *m/z* and thus cover a range of 50–2000 *m/z*. The collision energy for each window was determined according to the calculation for a charge 2+ ion centered upon the window with a spread of 15. Raw SWATH data files were converted into the mzXML format using ProteoWizard (version 3.0.3316)^55^ and data analysis was performed using the OpenSWATH tool^30^ integrated in the Euler portal workflow^56^. The OpenSWATH analysis workflow input files consisted of the mzXML files from the SWATH acquisitions, the TraML assay library file and the TraML file for iRT peptides. For the analysis of mid- and long- term DEN-treated mice cohorts (i.e. cohort 1and 2) we used input TraML assay library file that contained 3945 distinct protein groups (28′331 unique peptide sequences), while for NASH-induced HCC validation cohort 3 we enlarged initial assay library (i.e. 5222 distinct protein groups; 37′980 unique peptide sequences) by 25 additional DDA injections of new HCC samples in order to better fit the new analysis. All recorded SWATH data were extracted with 50 ppm around the expected mass of the fragment ions and with an extraction window of ±300 sec around the expected retention time after performing alignment of iRT peptides. The runs were subsequently aligned with a target FDR of 0.01 and a maximal FDR of 0.1 for aligned features. In the absence of a confidently identified feature, the peptide and protein intensities were obtained by integration of the respective background signal at the expected peptide retention time^31^. Next, the recorded feature intensities obtained from automatic OpenSWATH data processing were filtered with functions from the R/Bioconductor package SWATH2stats^45^ to reduce the size of the output data, remove low-quality features, and to only keep the features that were identified in at least 10% of data files. In fact, targeted SWATH analysis can search for specific peptides at a specific retention time and *m/z* ratio and input their requantification feature even if they are not highly abundant^31^. This function allowed us to quantify larger number of proteins among two different liver fractions, for all peptides confidently detected in at least in 10% of data files.

### Statistical data analysis

SWATH2stats filtered fragment intensities were introduced in the R/Bioconductor package MSstats (version MSstats.daily 2.3.5) and converted to protein abundances that were used for further statistical data analysis^57^. The quantification matrices of protein abundances measured in the lysate and mito data were obtained from MSstats analysis by using functions of data pre-processing, quality control of MS runs and model-based protein quantification per each biological sample. We define organelle enrichment scores as the measure of protein intracellular distribution with respect to the chosen organelle. Therefore, single mtFE protein score was calculated as difference of its relative abundances in the mitochondrion and total cellular lysate. We generated data matrices based on protein mtFE score values for all quantified proteins in three distinct mice cohorts. Quantification matrices were then used as input data template to perform further statistical analysis. One-way ANOVA and TukeyHSD post hoc analysis were used to detect a significant changes across four different liver conditions of discovery mice cohort (i.e. Ctrl-WT and Ctrl-KO, DEN-WT and DEN-KO). A two-dimensional centered heat map using R package “pheatmap” on the log-transformed, normalized relative protein intensities carried out hierarchical data clustering analysis. For hierarchical clustering of the scaled data, Manhattan distances were used. Volcano plots were generated in Rstudio with the function “volcano.plot”. The violin plots were plotted using the ggplot2 package. The Spearman correlation was calculated and visualized by using Corrgram package v.1.13 in Rstudio. Venn diagrams were drawn using an online tool (http://bioinformatics.psb.ugent.be/webtools/Venn/). The MitoCarta2.0 mouse inventory is a collection of 1158 nuclear and mtDNA genes encoding proteins with strong support of mitochondrial localization. This source was used to detect which of our identified proteins has strong evidence of mitochondrial localization.

### Data stratification in three datasets

Each protein data type (i.e., mtFE scores, lysate, and mito protein abundances) was analyzed separately in order to compare obtained protein classifiers that stratified healthy and cancer liver samples. All DEN-treated liver samples coming from either WT or Lrh-1 KO were labeled “Cancer” and compared with the group of control samples labeled “Control” coming from healthy mice (i.e., WT and LRH-1 KO, PBS-treated). In order to investigate the key proteins that can better distinguish between control and cancer samples based on their intensities or mtFE scores, we performed bioinformatics studies using two widely-employed machine-learning (ML) algorithms— Naïve Bayes (NB)^38^ and Logistic Regression (LR)^37^—with the popular machine-learning platform WEKA^58^. The pre-selection of differentially abundant proteins from three datasets with evidence of mitochondrion localization (based on MitoCarta) were used as input list for ML analysis. All the parameters for the model construction were implemented by default. Specifically, for each dataset, we extracted one protein each time to train and test the NB and LR models via five-fold cross-validation, due to the relatively small size of the training cohort (N = 28). We then ranked and shortlisted top 10 proteins based on their AUC values obtained by the two models. A final list of the overlap in the top 10 proteins (i.e., shortlisted by both algorithms) for each dataset together with their prediction performance is reported in Fig. 5.

The discovered protein candidates by using the ML approach in the original mice cohort 1 were individually verified in two distinct mouse model of liver cancer. In the first verification experiment we collected the liver samples of WT long term DEN-treated mice wherein each tumor contained their corresponding surrounding normal tissues. We applied paired t-test statistics to calculate mean difference between paired samples of tumors and controls with statistical probability (significant p < 0.05). In our final experiment we verified previously validated markers of DEN-induced carcinogenesis on the new NASH-induced mouse model of HCC by Student’s t-test applied between control and HCC mice group. For the final validated candidates we generated ROC curves in R using the pROC package^59^.

### Immunohistochemistry experiments

Liver tissue of long-term DEN-treated mice of both genotypes was fixed overnight in phosphate-buffered 10% formalin and embedded in paraffin, sectioned in 4 μm, and stained with eosin/hematoxylin. Immunohistochemistry was performed using anti-BrdU antibody (AbD Serotec, OBT0030) and anti-Ki67 (Abcam, Ab16667) antibodies. For 5-bromo-2′-deoxyuridine (BrdU, Sigma) incorporation, mice were intraperitoneally injected with BrdU at the dose of 100 mg/kg body weight for 4 hours before sacrifice. Liver section of NASH-induced HCC by CD-HFD in mice cohort 3 were stained with eosin/hematoxylin. Immunohistochemistry was performed using anti-GS antibody in dilution 1:500, purchased from Abcam (ab16802).

## Supplementary information

Supplementary Figures 1-7

Dataset 1

Dataset 2

Dataset 3

Dataset 4

Dataset 5

Dataset 6

Dataset 7

Dataset 8

Dataset 9

Dataset 10

## Data Availability

All the raw data of MS measurements, together with the input spectral library are available on the PRIDE archive (Discovery dataset identifier PXD008758, Reviewer account details: Username: reviewer53794@ebi.ac.uk Password: ozfE0NbW. Validation dataset identifier PXD013295, Reviewer account details: Username: reviewer42688@ebi.ac.uk Password: 6y46SurG.)
