# Supplementary material for: A new class of protein biomarkers based on subcellular distribution: application to a mouse liver cancer model
Source: Sci Rep. 2019 May 6;9:6913. doi: 10.1038/s41598-019-43091-z (PMC6502816; doi:10.1038/s41598-019-43091-z)
Supplement: Supplementary file 1 — Supplementary Figures 1-7 [file 41598_2019_43091_MOESM1_ESM.pdf]

**Supplementary Information:**

# A new class of protein biomarkers based on subcellular distribution: application to a mouse liver cancer model

Tatjana Sajic[1], Rodolfo Ciuffa[1], Vera Lemos[2], Pan Xu[2], Valentina Leone[3,4], Chen Li[1,5], Evan G. Williams[1], Georgios Makris[1], Amir Banaei-Esfahani[1,6], Mathias Heikenwalder[3], Kristina Schoonjans[2], Ruedi Aebersold[1,7*]

[1] Department of Biology, Institute of Molecular Systems Biology, ETH Zurich, Zurich, Switzerland

[2] Institute of Bioengineering, École Polytechnique Fédérale de Lausanne, Lausanne, Switzerland

[3] Division of Chronic Inflammation and Cancer, German Cancer Research Center (DKFZ), Heidelberg, Germany

[4] Research Unit Radiation Cytogenetics, Helmholtz Zentrum München Research Center for Environmental Health (GmbH), Neuherberg, Germany

[5]Biomedicine Discovery Institute and Department of Biochemistry and Molecular Biology, Monash University, Melbourne, VIC 3800, Australia

[6] PhD Program in Systems Biology, Life Science Zurich Graduate School, University of Zurich and ETH Zurich, Zurich, Switzerland

[7] Faculty of Science, University of Zurich, Zurich, Switzerland

*Corresponding author: aebersold@imsb.biol.ethz.ch

**Supplementary Figure 1.**

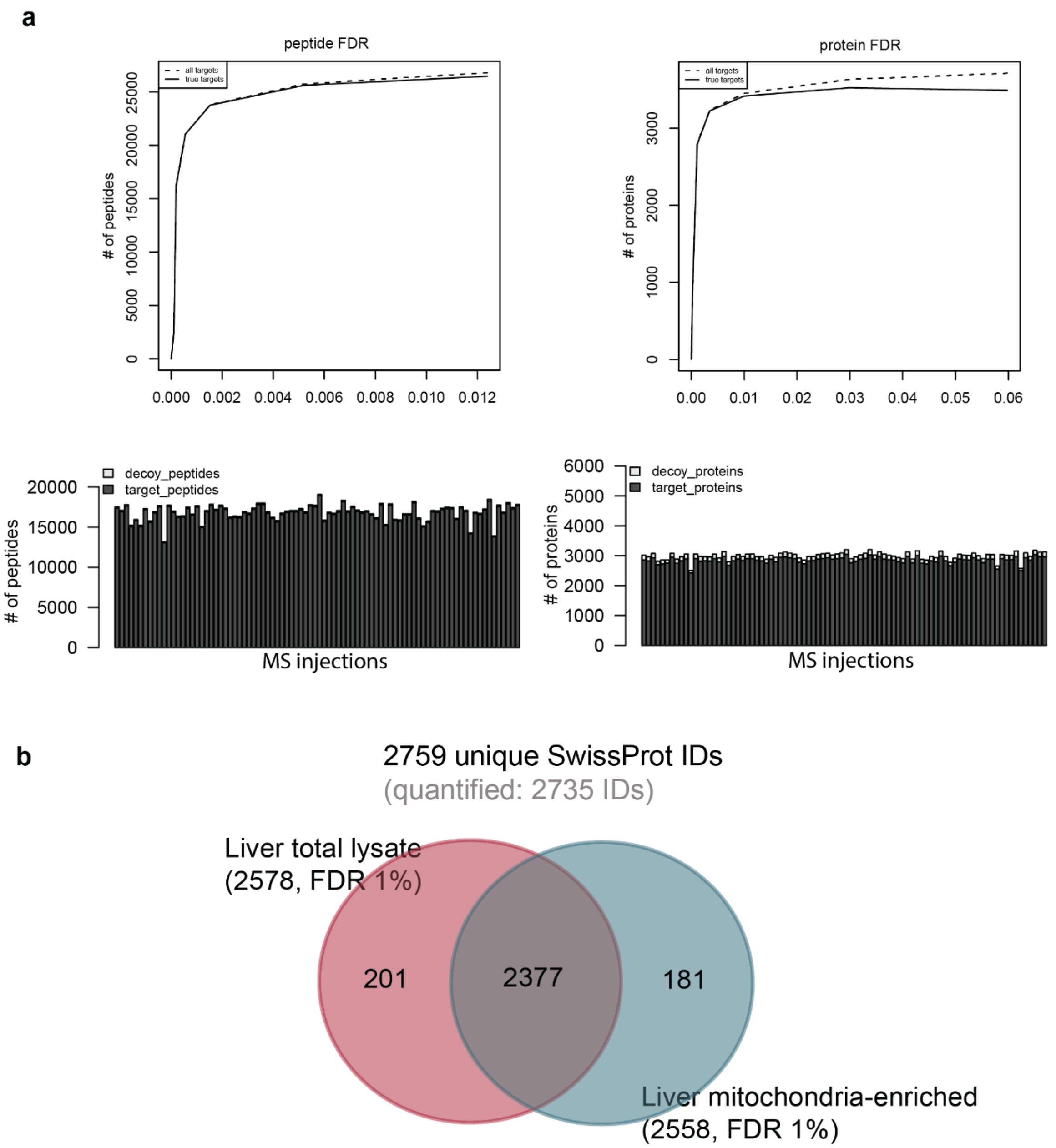


**Supplementary Figure 1: Reproducibility estimation at the peptide and protein level in the discovery cohort by SWATH2stats R-package.** (**a**) (Top) SWATH2stats global false discovery rate (FDR) report of OpenSWATH/pyProphet results. Receiver operating characteristics (ROC) curves of the estimation of FDR for number of identified peptides (left) and proteins (right). (Bottom) Barplot showing the stable and consistent peptides and proteins identification across all samples and replicates. (**b**) Venn diagram is showing the intersection of the proteins identified at 1% of protein FDR in total lysate and mitochondria-enriched fraction, respectively. The q-value threshold on level of OpenSWATH peptide query scores was used to assess the protein FDR.

**Supplementary Figure 2.**

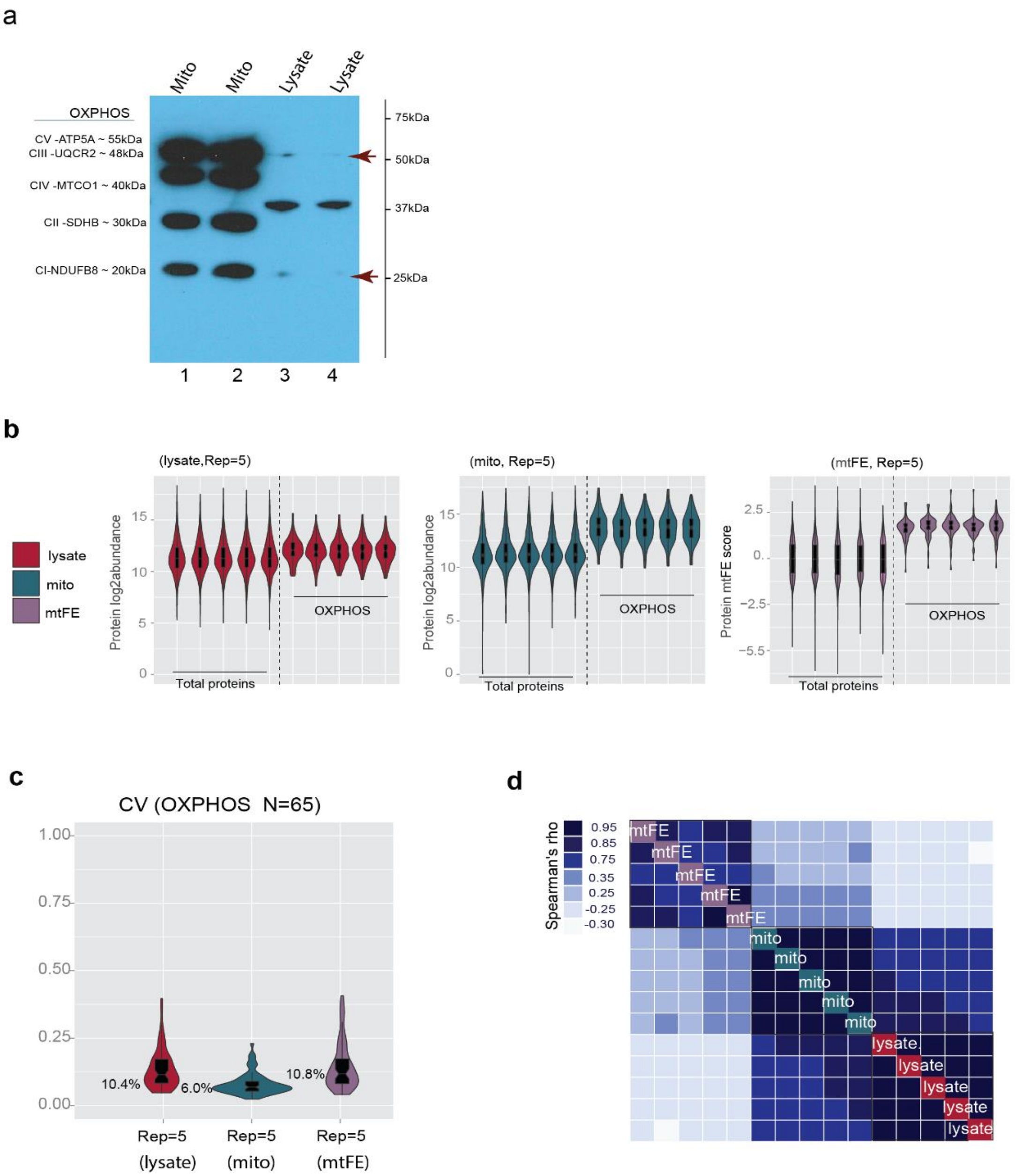


**Supplementary Figure 2: Control of data quality.** (**a**) Western Blot detection of five OXPHOS enzymes in mitochondrial fraction (lanes 1 and 2) and in cellular lysate (lanes 3 and 4). After long membrane exposure (t=1 min) by chemiluminescence, the bands of CI subunit NDUFB8 , CII-35KDa, and CV alpha subunit-53 KDa of OXPHOS enzymes appear on the lines corresponding to total cellular lysate that also contain mitochondria. (**b**) OXPHOS protein distribution across three different data readouts of replicated samples. (**c**) CVs (Coefficient of variation) calculated for statically localized OXPHOS enzymes in the enriched mitochondrial fraction, between five replicates of lysate, mito, and mtFE data. The replicates correspond to five repeated liver preparations of cellular lysate, mitochondria-enriched, and calculated mtFE data. (**d**) Spearman's rank correlation coefficients of protein log2 intensities in replicates of total lysate, enriched-mitochondria, and calculated mtES scores compared against each other. Black boxes highlight comparisons between five replicates ordered by strength of correlation. mtES protein scores, calculated from the lysate and enriched-mitochondrial fraction for each of the five respective replicates, present high range of Spearman's rho correlation of 0.75-0.90.

**Supplementary Figure 3.**

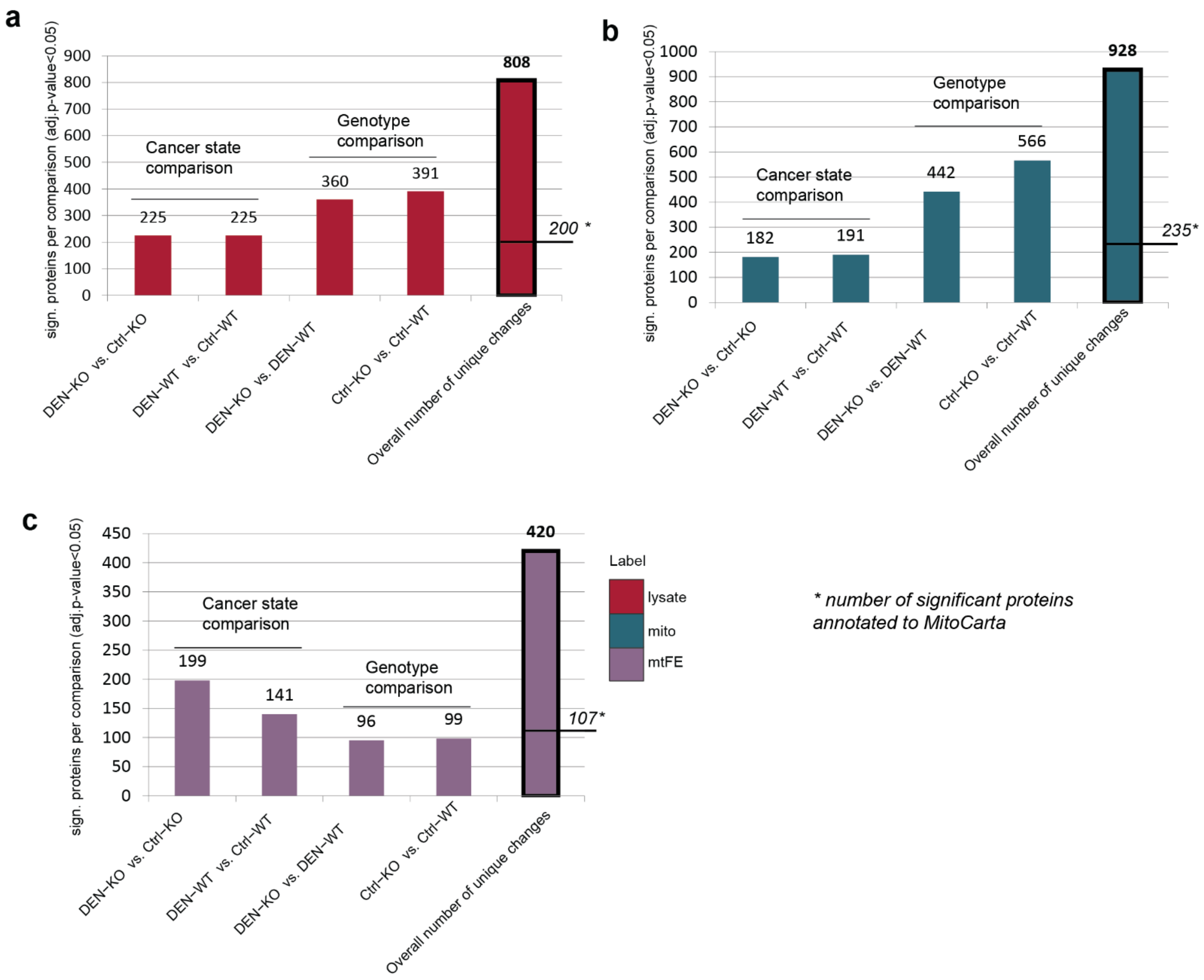


**Supplementary Figure 3: One-way ANOVA comparisons of liver conditions in lysate, mito, and mtFE scores data (a-c)**. The number of ANOVA significant proteins of four distinct comparisons (i.e., comp1=Ctrl-WT vs Ctrl-KO, comp2= DEN-WT vs DEN-KO, comp3= Ctrl-WTvs DEN-WT, and comp4= Ctrl-KO vs DEN-KO) performed on each data set are presented by bar plots. The bar plots are shown by different color codes, corresponding to three respective datasets: lysate (dark red), mito (dark green) and mtFE (dark violet).

## Supplementary Figure 4.

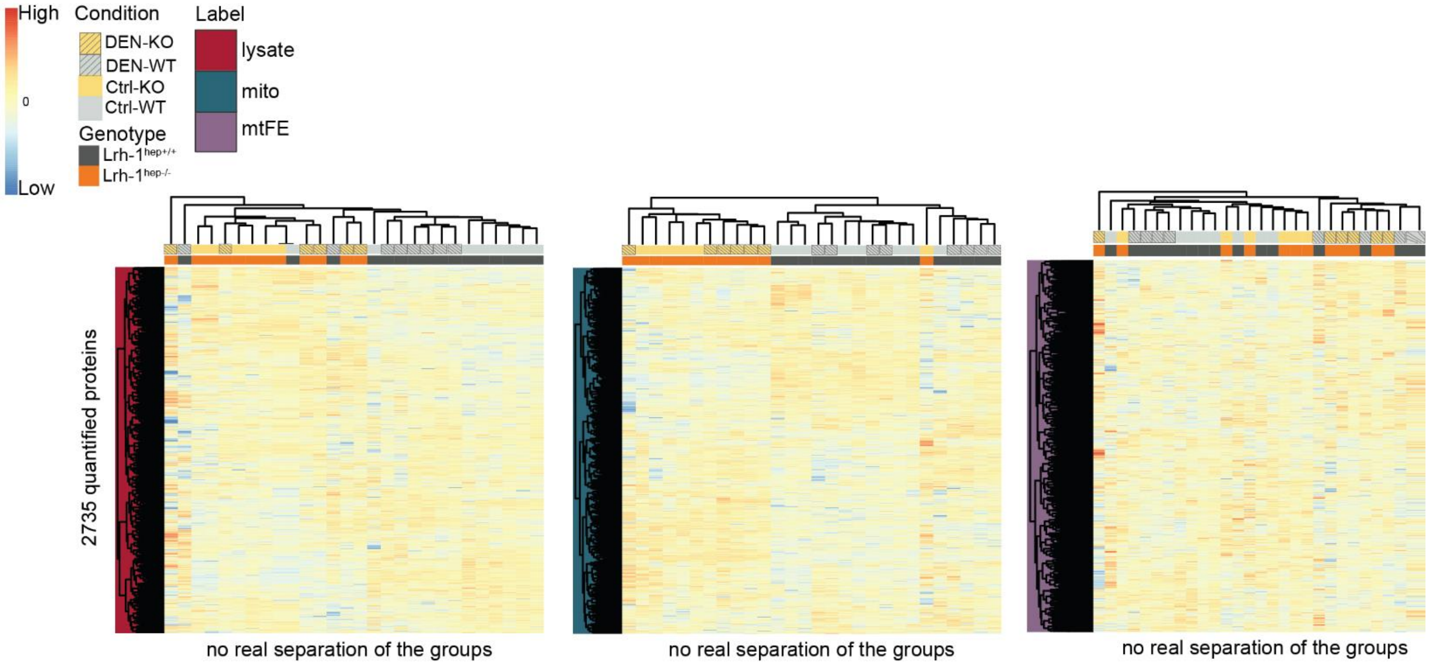


**Supplementary Figure 4**: **Unsupervised hierarchical data clustering.** Unsupervised hierarchical clustering presents three heat-maps with no real separation of the groups among 28 samples. The based on the 2735 proteins, quantified as abundances in the total lysate and enriched-mitochondrion, and as calculated scores in mtFE data.

## Supplementary Figure 5.

**a** *Naïve Bayes-Based Stratification (discovery cohort)*

Cellular lystae (cohort 1)

| UniProt | Gene Name | AUC | Accuracy |
|---|---|---|---|
| **Q8CGK3** | **Lonp1** | 0.99 | 0.96 |
| **P62270** | **Rps18** | 0.93 | 0.96 |
| **Q9CPQ8** | **Atp5l** | 0.92 | 0.89 |
| **P61922** | **Abat** | 0.91 | 0.93 |
| **Q91VD9** | **Ndufs1** | 0.91 | 0.82 |
| **Q9CR62** | **Slc25a11** | 0.91 | 0.89 |
| **Q9D0R2** | **Tars** | 0.91 | 0.79 |
| **Q9DCT2** | **Ndufs3** | 0.91 | 0.89 |
| **P08228** | **Sod1** | 0.90 | 0.93 |
| P62264 | Rps14 | 0.90 | 0.86 |

Mitochondrial lysate (cohort 1)

| UniProt | Gene Name | AUC | Accuracy |
|---|---|---|---|
| **Q8K354** | **Cbr3** | 0.95 | 0.82 |
| **P47199** | **Cryz** | 0.92 | 0.75 |
| **P56393** | **Cox7b** | 0.92 | 0.86 |
| **Q9CXZ1** | **Ndufs4** | 0.90 | 0.82 |
| **Q9CQZ6** | **Ndufb3** | 0.89 | 0.79 |
| **Q9CQJ8** | **Ndufb9** | 0.88 | 0.82 |
| **Q9CQ75** | **Ndufa2** | 0.87 | 0.82 |
| **Q9D0S9** | **Hint2** | 0.87 | 0.82 |
| **Q9D6J6** | **Ndufv2** | 0.86 | 0.82 |
| Q8QZS1 | Hibch | 0.86 | 0.75 |

mtES (cohort 1)

| UniProt | Gene Name | AUC | Accuracy |
|---|---|---|---|
| **Q8CGK3** | **Lonp1** | 0.93 | 0.86 |
| **P08228** | **Sod1** | 0.93 | 0.93 |
| **Q9CR62** | **Slc25a11** | 0.92 | 0.89 |
| **P97493** | **Txn2** | 0.91 | 0.86 |
| **Q60597** | **Ogdh** | 0.91 | 0.89 |
| **P61922** | **Abat** | 0.90 | 0.79 |
| **Q3U5Q7** | **Cmpk2** | 0.89 | 0.75 |
| **Q8VE22** | **Mrps23** | 0.89 | 0.75 |
| **Q9WVM8** | **Aadat** | 0.88 | 0.82 |
| Q80XL6 | Acad11 | 0.87 | 0.86 |

*UniProt name in bold overlap with predictors discovered by Logistic Regression-Based model

**b** *Validation cohort long term DEN, WT mice (n=10 liver samples)*

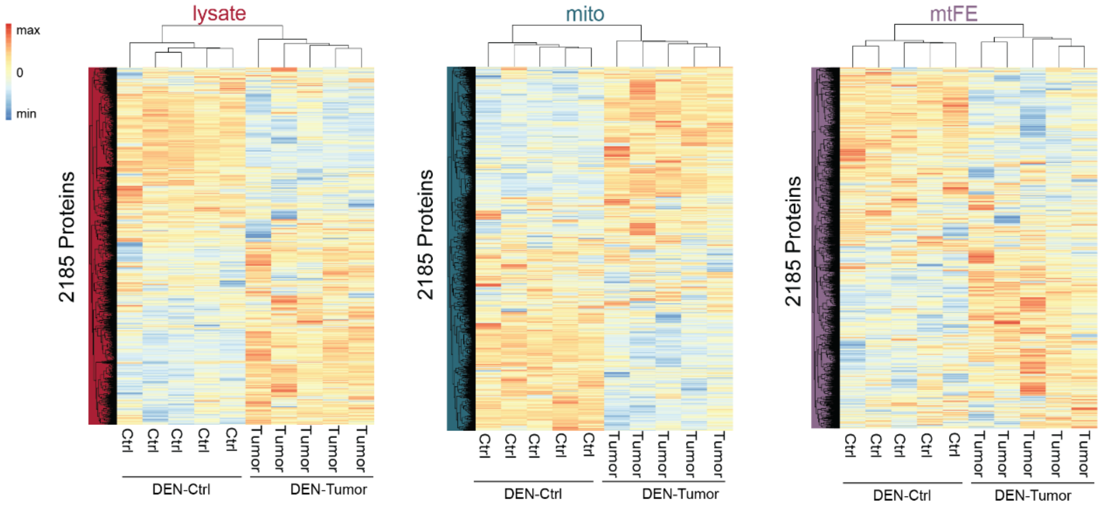


**c.** *Summary statistics of individual ROCs (validation cohort)*

lysate

**Rps18**

| Cohort2 | (CI 95%) |
|---|---|
| AUC | 1.0 (1.0–1.0) |
| Sensitivity | 1.0 (1.0–1.0) |
| Specificity | 1.0 (1.0–1.0) |
| Accuracy | 1.0 (1.0–1.0) |

**Abat**

| Cohort2 | (CI 95%) |
|---|---|
| AUC | 1.0 (1.0–1.0) |
| Sensitivity | 1.0 (1.0–1.0) |
| Specificity | 1.0 (1.0–1.0) |
| Accuracy | 1.0 (1.0–1.0) |

**Ndufs3**

| Cohort2 | (CI 95%) |
|---|---|
| AUC | 1.0 (1.0–1.0) |
| Sensitivity | 1.0 (1.0–1.0) |
| Specificity | 1.0 (1.0–1.0) |
| Accuracy | 1.0 (1.0–1.0) |

**Ndufs1**

| Cohort2 | (CI 95%) |
|---|---|
| AUC | 1.0 (1.0–1.0) |
| Sensitivity | 1.0 (1.0–1.0) |
| Specificity | 1.0 (1.0–1.0) |
| Accuracy | 1.0 (1.0–1.0) |

**Slc25a11**

| Cohort2 | (CI 95%) |
|---|---|
| AUC | 0.88 (0.62–1.0) |
| Sensitivity | 0.80 (0.40-1.0) |
| Specificity | 1.0 (1.0-1.0) |
| Accuracy | 0.90(0.83-1.0) |

mito

**Ndufv2**

| Cohort2 | (CI 95%) |
|---|---|
| AUC | 1.0 (1.0–1.0) |
| Sensitivity | 1.0 (1.0–1.0) |
| Specificity | 1.0 (1.0–1.0) |
| Accuracy | 1.0 (1.0–1.0) |

**Ndufs4**

| Cohort2 | (CI 95%) |
|---|---|
| AUC | 1.0 (1.0–1.0) |
| Sensitivity | 1.0 (1.0–1.0) |
| Specificity | 1.0 (1.0–1.0) |
| Accuracy | 1.0 (1.0–1.0) |

**Ndufb3**

| Cohort2 | (CI 95%) |
|---|---|
| AUC | 0.96 (0.85–01.0) |
| Sensitivity | 1.0 (1.0–1.0) |
| Specificity | 0.80 (0.40-1.0) |
| Accuracy | 0.90 (0.83-1.0) |

**Ndufb9**

| Cohort2 | (CI 95%) |
|---|---|
| AUC | 1.0 (1.0–1.0) |
| Sensitivity | 1.0 (1.0–1.0) |
| Specificity | 1.0 (1.0–1.0) |
| Accuracy | 1.0 (1.0–1.0) |

mtFE

**Lonp1**

| Cohort2 | (CI 95%) |
|---|---|
| AUC | 0.88 (0.62–1.0) |
| Sensitivity | 0.80 (0.40-1.0) |
| Specificity | 1.0 (1.0–1.0) |
| Accuracy | 0.90 (0.83-1.0) |

**Tnx2**

| Cohort2 | (CI 95%) |
|---|---|
| AUC | 1.0 (1.0–1.0) |
| Sensitivity | 1.0 (1.0–1.0) |
| Specificity | 1.0 (1.0–1.0) |
| Accuracy | 1.0 (1.0–1.0) |

**Ogdh**

| Cohort2 | (CI 95%) |
|---|---|
| AUC | 0.88 (0.62–1.0) |
| Sensitivity | 0.80 (0.40-1.0) |
| Specificity | 1.0 (1.0–1.0) |
| Accuracy | 0.90 (0.83-1.0) |

**Cmpk2**

| Cohort2 | (CI 95%) |
|---|---|
| AUC | 1.0 (1.0–1.0) |
| Sensitivity | 1.0 (1.0–1.0) |
| Specificity | 1.0 (1.0–1.0) |
| Accuracy | 1.0 (1.0–1.0) |

**Mrps23**

| Cohort2 | (CI 95%) |
|---|---|
| AUC | 1.0 (1.0–1.0) |
| Sensitivity | 1.0 (1.0–1.0) |
| Specificity | 1.0 (1.0–1.0) |
| Accuracy | 1.0 (1.0–1.0) |

**d.**

Common quantified proteins in dicovery and validation cohort

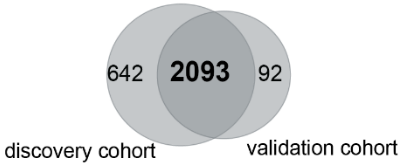


Common differentially expressed proteins in discovery and validation cohort

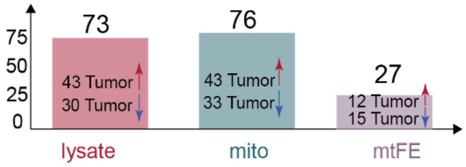

**Supplementary Figure 5: Protein biomarkers of three data types in DEN-induced liver carcinogenesis.** (**a**) Top selected tissue markers based on Naïve Bayes (NB) analysis. Three protein panels present top-selected markers ranked according to their average AUC value for three respective datasets: lysate (left panel), mito (mid panel) and mtFE data (right panel). (**b**) Heat map visualization of data clustering based on 2185 proteins quantified in the validation cohort of long-term DEN-treated WT liver. (**c**) Validation cohort summary statistics (i.e., specificity, sensitivity, and accuracy at 95% CI) of individual tissue-markers were calculated at an optimal cutoff point as determined by Youden's index (Youden, 1950) for each of the three respective datasets. Optimistic values for specificity, sensitivity, and accuracy at 95% CI in the small sample size available would be expected to be of inferior value in a cohort of large sample (**d**) Venn Diagram presents common quantified proteins across discovery and validation experiment. The bar plot shown by different color codes, corresponding to three respective datasets, (i.e. lysate (dark red), mito (dark green) and mtFE (dark violet)) present the number of common regulated proteins across discovery and validation cohort. Dark blue or dark red color of arrow corresponds to protein number with decreased or increased abundance in tumor respectively.

## Supplementary Figure 6.

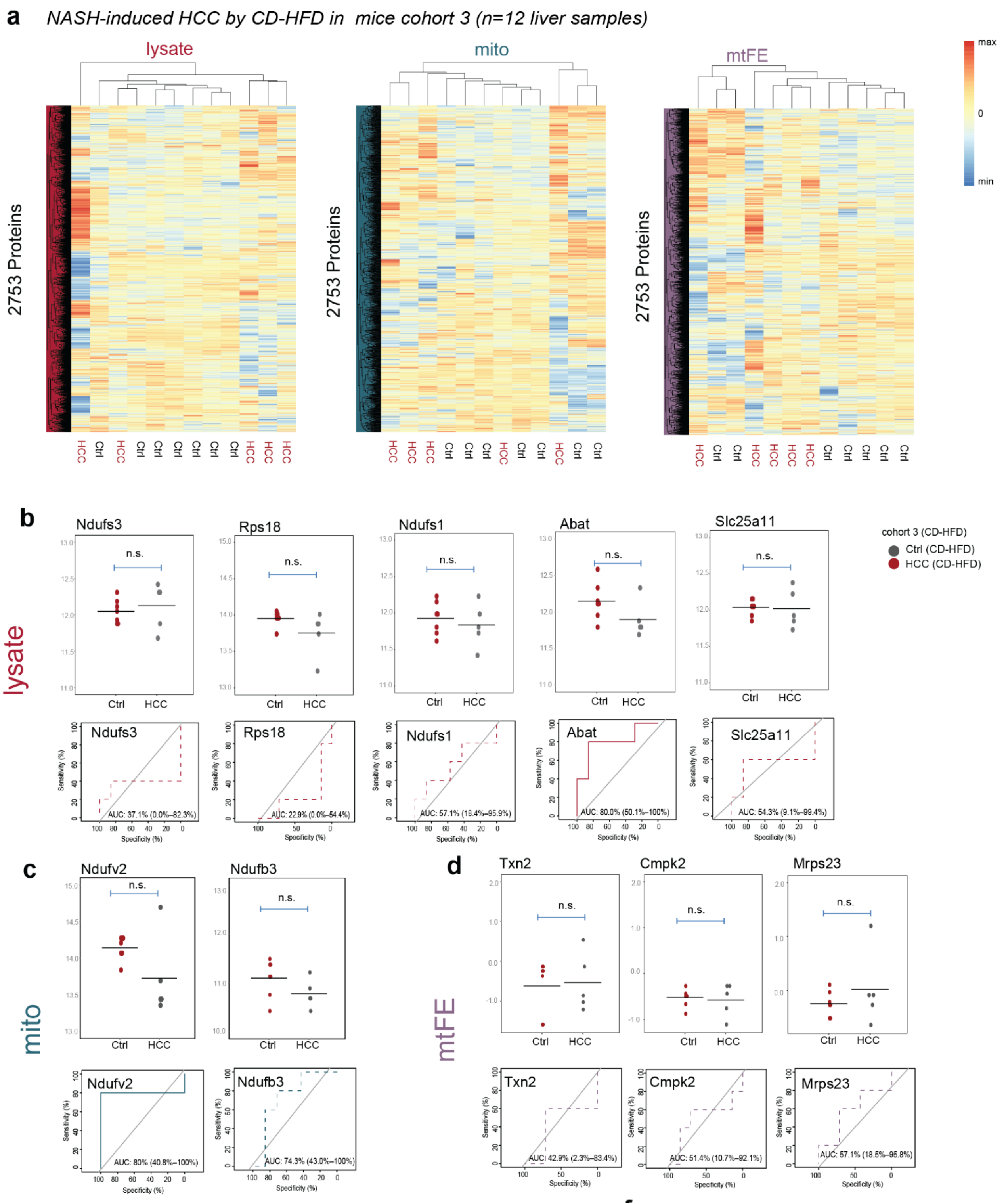


**e** *Summary statistics of individual ROCs (cohort 3)*

mito

**Ndufs4**

| Cohort3 | (CI 95%) |
|---|---|
| AUC | 0.77 (0.39–1.0) |
| Sensitivity | 0.80 (0.40–1.0) |
| Specificity | 0.86 (0.57–1.0) |
| Accuracy | 0.83 (0.66-1.0) |

**Ndufb9**

| Cohort3 | (CI 95%) |
|---|---|
| AUC | 0.87 (0.65-1.0) |
| Sensitivity | 0.80 (0.5–1.0) |
| Specificity | 1.0 (1.0–1.0) |
| Accuracy | 0.92 (0.67–1.0) |

mtFE

**Lonp1**

| Cohort3 | (CI 95%) |
|---|---|
| AUC | 0.86(0.63–1.0) |
| Sensitivity | 0.60 (0.20–1.0) |
| Specificity | 1.0 (0.43–1.0) |
| Accuracy | 0.83 (0.66-1.0) |

**Ogdh**

| Cohort3 | (CI 95%) |
|---|---|
| AUC | 0.83 (0.54–1.0) |
| Sensitivity | 0.80 (0.40–1.0) |
| Specificity | 0.86 (0.57–1.0) |
| Accuracy | 0.83 (0.66-1.0) |

**f**

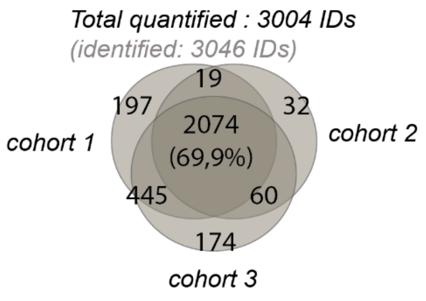

**Supplementary Figure 6: Lysate, mito and mtFE scores proteome data in the NASH-induced model of HCC**. (**a**) Heat map visualization of data clustering based on proteins quantified in NASH-induced HCC mice cohort 3. Three heat maps correspond to respective datasets: lysate (dark red), mito (dark green) and mtFE (dark violet). (**b-e**) Dot plots and ROC curves of corresponding lysate, mito and mtFE markers that were not significantly dysregulated in the new mice model of HCC (**b-d**). (**e**) Summary statistics (i.e., specificity, sensitivity, and accuracy at 95% CI calculated with an optimal cutoff point as determined by Youden's index) of confirmed protein markers calculated in the mice cohort 3 for two respective datasets. (**e**) Venn diagram results represent the number of shared proteins that were quantified between mice cohorts. Of total 3004 quantified proteins, around 70 % were quantified and analyzed over different conditions/ mouse models of liver cancer.

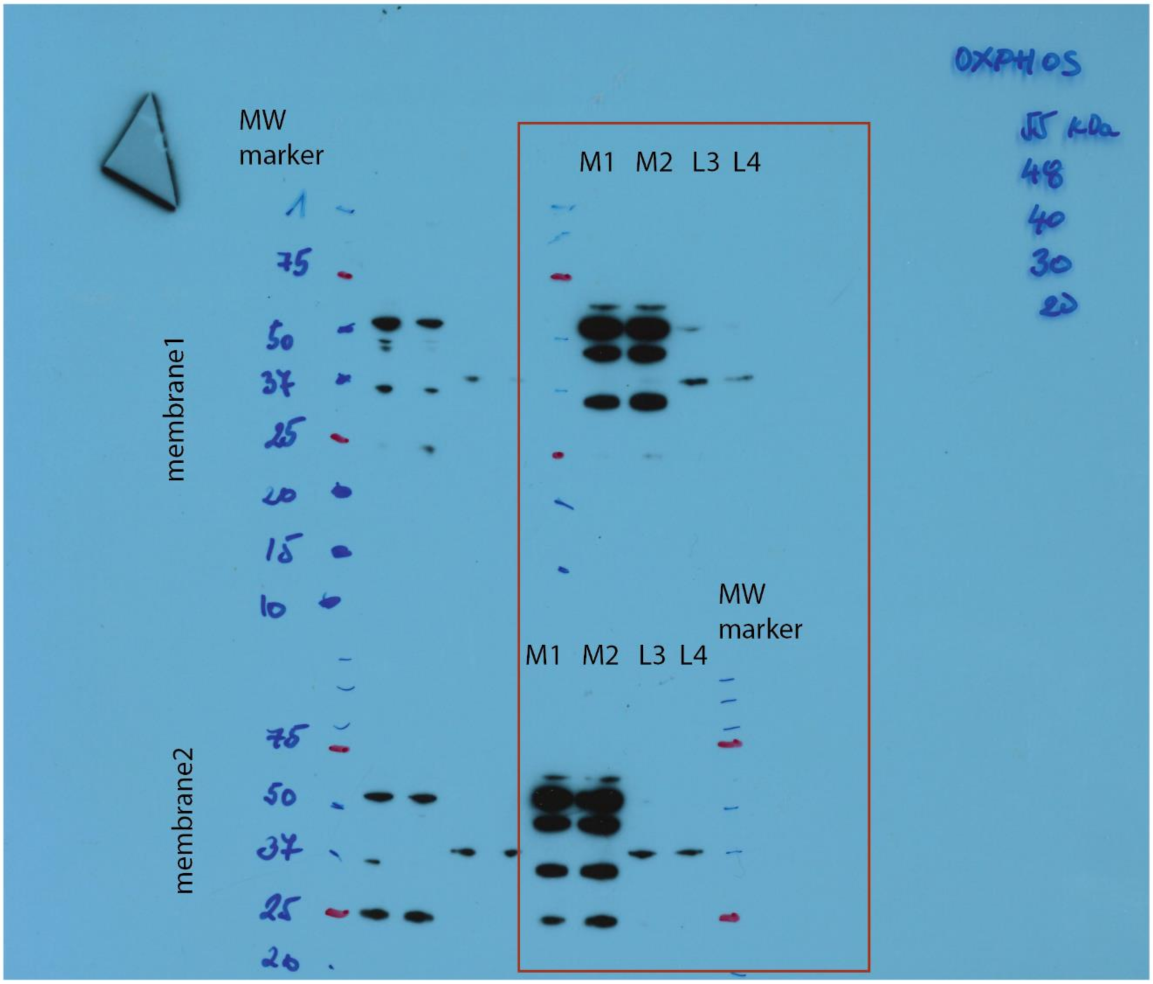


**Supplementary Figure 7: Full membranes detected for Western blots shown in the main and supplementary figures.** Upper and lower membrane (i.e. membrane 1 and 2) are corresponding to experimental replicates of mitochondria-enriched and total lysate isolation by two different protocols. In the right red square, the samples were isolated by using a differential centrifugation steps in the sucrose density solution (200mM) as described in the main text. The representative picture in the main Figure 2 corresponds to the samples detected on the membrane 2 in the red square. The protein samples were in reduced state during gel electrophoresis. An antibody cocktail contains Anti-NDUFB8 (~20KD), Anti-SDHB (~30KD), Anti-UQCRC2 (~48Kgel D), Anti-MTCO1 (~40KD), AntiATP5A1 (~55KD) was used.
